# BCR-ABL Affects STAT5A and STAT5B Differentially

**DOI:** 10.1371/journal.pone.0097243

**Published:** 2014-05-16

**Authors:** Michael Schaller-Schönitz, David Barzan, Andrew J. K. Williamson, John R. Griffiths, Iris Dallmann, Karin Battmer, Arnold Ganser, Anthony D. Whetton, Michaela Scherr, Matthias Eder

**Affiliations:** 1 Hannover Medical School, Department of Hematology, Hemostasis, Oncology and Stem Cell Transplantation, Hannover, Germany; 2 Faculty of Medical and Human Sciences, Manchester Academic Health Science Centre, University of Manchester, Wolfson Molecular Imaging Centre, Manchester, United Kingdom; Emory University, United States of America

## Abstract

Signal transducers and activators of transcription (STATs) are latent cytoplasmic transcription factors linking extracellular signals to target gene transcription. Hematopoietic cells express two highly conserved STAT5-isoforms (STAT5A/STAT5B), and STAT5 is directly activated by JAK2 downstream of several cytokine receptors and the oncogenic BCR-ABL tyrosine kinase. Using an IL-3-dependent cell line with inducible BCR-ABL-expression we compared STAT5-activation by IL-3 and BCR-ABL in a STAT5-isoform specific manner. RNAi targeting of STAT5B strongly inhibits BCR-ABL-dependent cell proliferation, and STAT5B but not STAT5A is essential for BCL-X_L_-expression in the presence of BCR-ABL. Although BCR-ABL induces STAT5-tyrosine phosphorylation independent of JAK2-kinase activity, BCR-ABL is less efficient in inducing active STAT5A:STAT5B-heterodimerization than IL-3, leaving constitutive STAT5A and STAT5B-homodimerization unaffected. In comparison to IL-3, nuclear accumulation of a STAT5A-eGFP fusion protein is reduced by BCR-ABL, and BCR-ABL tyrosine kinase activity induces STAT5A-eGFP translocation to the cell membrane and co-localization with the IL-3 receptor. Furthermore, BCR-ABL-dependent phosphorylation of Y682 in STAT5A was detected by mass-spectrometry. Finally, RNAi targeting STAT5B but not STAT5A sensitizes human BCR-ABL-positive cell lines to imatinib-treatment. These data demonstrate differences between IL-3 and BCR-ABL-mediated STAT5-activation and isoform-specific effects, indicating therapeutic options for isoform-specific STAT5-inhibition in BCR-ABL-positive leukemia.

## Introduction

Signal transducers and activators of transcription (STATs) are a family of proteins involved in signal transduction from multiple cytokine or growth factor receptors with a similar modular composition (STAT1, 2, 3, 4, 5A, 5B, and 6) [1], [2]. Inactive STATs are believed to exist either as monomers or pre-formed dimers in an anti-parallel conformation. Upon receptor activation STATs are recruited to activated receptors, and tyrosine phosphorylation of a critical C-terminal residue leads to dimerization or to conformational changes of pre-formed dimers into a parallel orientation involving reciprocal phosphotyrosine-SH2-domain interactions. Active dimers translocate to the nucleus and initiate target gene transcription which may occur via tetrameric STAT-complexes [3].

STAT5 has a critical role within the hematopoietic system: it is activated by the receptors for Epo, GM-CSF, G-CSF, TPO, IL-2, IL-3, IL-5, IL-7, and IL-15 [1], [4]. STAT5 exists in two isoforms with high sequence homology, STAT5A and STAT5B, which are encoded by two different genes. Generation of STAT5A and/or STAT5B null mice has demonstrated redundant and differential functions for these 2 isoforms in predominantly non-hematopoietic cells [5]–[8]. However, development, proliferation and differentiation of hematopoietic progenitors are also affected by inactivation of STAT5 genes [9]–[12]. STAT5 activation appears to involve a similar molecular event including phosphorylation of Y694 (STAT5A) and Y699 (STAT5B) [13]. Substitution of Y694 and Y699 with phenylalanine results in dominant-negative STAT5 mutants which inhibit cell proliferation and induce apoptosis. One well characterized STAT5 target is the anti-apoptotic BCL-X_L_ gene essential for fetal erythropoiesis [14]–[16].

The oncogenic fusion gene BCR-ABL results from the reciprocal translocation t(9;22)(q34;q22) characteristic for chronic myeloid leukemia (CML) and BCR-ABL-positive acute lymphoblastic leukemia (ALL). BCR-ABL is a constitutively active cytoplasmic tyrosine kinase which activates many intracellular signalling cascades largely overlapping with those activated by cytokine receptors [17]–[19]. STAT5 is activated by BCR-ABL and is required for induction and maintenance of BCR-ABL-positive leukemia in mice [20]–[22]. However, we have shown that BCR-ABL is less effective than cytokines to induce proliferation of cells with reduced STAT5 expression using an RNAi-approach targeting STAT5A and STAT5B simultaneously [23].

To compare STAT5 activation by IL-3 with that by BCR-ABL we used the TonB cell line with inducible BCR-ABL-expression and analyzed STAT5A- and STAT5B-specific loss- and gain-of function phenotypes in the presence and absence of IL-3 and BCR-ABL. This approach allows direct comparison of IL-3 function with that of BCR-ABL under identical expression and stoichiometry of STAT5-isoforms. We show tyrosine phosphorylation of STAT5 by BCR-ABL independent of JAK2 kinase activity and reduced activation of STAT5A by BCR-ABL as compared to IL-3. BCR-ABL affects STAT5A:STAT5B-heterodi-(oligo)merization, intracellular localization of STAT5A and induces aberrant phosphorylation of Y682 in STAT5A as determined by mass spectrometry. These data provide molecular evidence for differences in STAT5 activation by the IL-3 receptor (IL-3R)/JAK2 and by BCR-ABL, isoform-specific effects of BCR-ABL on STAT5 and a new system of regulation in STAT5A. They may also indicate a yet unknown therapeutic option for STAT5-inhibition in BCR-ABL-positive leukemia.

## Materials and Methods

### Cell Culture

TonB cells were cultured in RPMI 1640 supplemented with 10% [v/v] FCS (Biochrome, Berlin, Germany) and 10–15% [v/v] WEHI-3B conditioned media (WEHI3B-CM) as a source of murine IL-3. Expression of p210BCR-ABL was induced by addition of doxycycline [1.5 µg/mL] which was replaced every three days. EM-2, K562 and LAMA-84 cells were cultured in RPMI 1640 with 10% [v/v] FCS (Biochrome, Berlin, Germany). CD34+ cells from healthy volunteers and CML patients were obtained after written informed consent has been obtained. The study was approved by the local Ethics Committee of Hannover Medical School.

### shRNA Synthesis

Construction and evaluation of isoform-specific STAT5 shRNAs were performed as previously described [23], [24]. For detailed information see Information S1.

### Construction of Lentiviral Vectors

pCMV-SPORT6 plasmids encoding murine STAT5A (IRAVp968G0222D) and STAT5B (IRAVp968D0246D) cDNAs were purchased from Source BioScience imaGenes (Berlin, Germany). STAT5-cDNAs were excised with 5′*Sal*I and 3′*Hin*dIII (STAT5A) and 5′*Sal*I and 3′*Spe*I (STAT5B), blunted and inserted into *Bam*HI-digested pHR-SIN-SIEW-*Sna*BI [25].

A STAT5A-eGFP expression plasmid (pN1-S5AeGFP) was kindly provided by Hansjörg Hauser and Mario Köster (Helmholtz Centre for Infection Research, Braunschweig, Germany). To insert the STAT5A-eGFP cassette into the lentiviral pHR-SIN-SR-*SnaB*I vector [26] a *Kpn*I restriction site was created upstream the RFP gene by site-directed mutagenesis (according to the manufacturer guidelines, Stratagene, La Jolla, CA). Murine STAT5A-eGFP was excised with 5′*Bgl*II and 3′*Kpn*I and inserted into 5′*Bam*HI/3′*Kpn*I-digested pHR-SIN-SR-*SnaB*I. This strategy resulted in a loss of RFP from the lentiviral transgene plasmid.

For lentiviral expression of shRNAs the H1-shRNA cassettes were cloned into the U3 region of the Δ3′ long terminal repeat (LTR) of pHR-SIN-SR-*SnaB*I as described before [24].

For construction of epitope-tagged STAT5 isoforms see Information S1.

All molecular modifications were verified by control digestions or DNA sequencing. The preparation of recombinant lentiviral supernatants and lentiviral transductions were performed as described earlier [23].

### Proliferation Assay

Cell proliferation was analyzed by the Trypan-blue dye exclusion assay or the MTS Assay. For the Trypan-blue dye exclusion assay, TonB cells were cultured in 24-well plates as follows: 1×10^4^ cells/mL in the presence of IL-3 and/or BCR-ABL, and 3×10^4^ cells/mL in the absence of IL-3 (Starvation). Human cell lines were cultured at 1.5×10^4^ cells/mL in 24-well plates and the number of viable cells was determined 96 hours later by Trypan-blue exclusion. For the MTS Assay TonB cells were cultured for 48 hours with Ruxolitinib and proliferation was measured using MTS (Promega, Mannheim, Germany). Therefore 20 µL of MTS substrate was added to 200 µL cell suspension in a 96 well plate, incubated for 3–4 hours at 37°C in a humidified, 5% CO_2_ atmosphere and absorbance was recorded at 490 nm using an ELISA plate reader (Mithras LB 940, Berthold Technologies, Bad Wildbad, Germany).

### Immunoblotting and Immunoprecipitation

TonB cells cultured with IL-3 or Dox (BCR-ABL) were either starved by removal of IL-3 or BCR-ABL tyrosine kinase activity was inhibited by addition of 1 µM imatinib mesylate (IM) overnight. Whole cell lysates were prepared with either RIPA buffer (50 mM Tris-HCl, pH 7.5; 150 mM NaCl; 1% Triton X-100; 0.5% sodium-deoxycholate; 0.1% SDS; 5 mM EDTA) supplemented with appropriate inhibitors (25 mM NaF; 5 mM Na_3_VO_4_; 10 mM AEBSF; 25 µM ALLN; 40 µM MG-132; 20 µM Protein Tyrosine Phosphatase Inhibitor Set IV and 1/50^th^ volume of Protease Inhibitor Cocktail Set III - all purchased from Calbiochem, La Jolla, CA, USA) or by direct lysis in NuPAGE LDS Sample Buffer (Invitrogen, Karlsruhe, Germany).

For preparation of subcellular extracts see Information S1.

Protein concentrations were determined using the Bradford protein assay (Bio-Rad, Munich, Germany).

Immunoprecipitations (normally 2–3 mg of total protein) were performed overnight in RIPA buffer supplemented with inhibitors, specific antibodies (3–5 µg) and Protein A/G Plus-Agarose Beads (Santa Cruz Biotechnology, Heidelberg, Germany). Proteins were denatured by resuspension in NuPAGE LDS Sample Buffer plus NuPAGE Reducing Agent (Invitrogen, Karlsruhe, Germany) followed by incubation for 10 minutes at 95°C. Lysates were separated by sodium dodecyl sulfate-polyacrylamide gel electrophoresis (SDS-PAGE) and transferred to Optitran BA-S 83 reinforced nitrocellulose membranes (Whatman, Dassel, Germany). Membranes were incubated with following antibodies: STAT5A (L-20), STAT5B (G-2), pTyr (PY99), ERK2 (C-14), anti-mouse-IgG-HRP, anti-rabbit-IgG-HRP from Santa Cruz Biotechnology; α-Tubulin (DM1A) from Calbiochem; STAT5A (611835), BCL-x (610211) from BD Transduction; STAT5B from BD Pharmingen (556517) or from R&D Systems (AF1584) and phospho-STAT5 (Tyr694) (C11C5), phospho-JAK2 (Tyr1007/1008) (C80C3), phospho-GAB2 (Tyr452) (C33G1), BCL-2 (#2876), HA-Tag (6E2), MYC-Tag (9B11), and β-Actin (13E5) from Cell Signaling.

Visualization was obtained by chemiluminescence using the ECL Western Blotting Detection Reagents (GE Healthcare, Munich, Germany). Densitometry was performed using a VersaDoc-4000MP imaging system equipped with QuantityOne quantification software (Bio-Rad, Munich, Germany).

### Immunofluorescence

TonB cells were transferred to glass slides by spin-occulation and fixed in 4% [w/v] PFA for 10 minutes. Permeabilization was performed with 0.05% Triton X-100 for 4 minutes. Cells were blocked in PBS supplemented with 10% [v/v] FCS (Biochrome, Berlin, Germany) for 1 hour and stained with PE Rat anti-mouse CD131 (559920) from BD Pharmingen and DAPI (0.5 µg/mL) for 2 hours. Samples were embedded in Mowiol plus DABCO (Roth, Karlsruhe, Germany).

Intracellular localization of STAT5AeGFP-expressing TonB cells was analysed in living cells. Cells were concentrated by centrifugation and resuspended in a small volume of medium. Cells were transferred to poly-L-lysine-coated glass slides and covered with poly-L-lysine-coated cover slides. Both were sealed with silicone and analyzed immediately.

Microscopic analyses were run on a Leica DM IRB laser scanning microscope equipped with a TCS SP2 AOBS scan head, a 405 nm light source for excitation of blue dyes and Leica LCS Lite software (Leica, Wetzlar, Germany).

### Selected Reaction Monitoring of STAT5 Isoforms

For MS/MS analysis STAT5 isoforms were immunoprecipitated in larger scales. At least 25 mg of total protein were precipitated with either 50 µg STAT5A (L-20) or STAT5B (G-2) antibodies, then resolved by SDS-PAGE and visualized using a colloidal Coomassie staining (2% [v/v] phosphoric acid, 5% [w/v] Al_2_(SO_4_)_3_×(H_2_O)_17_, 10% [v/v] ethanol, and 0.05% Coomassie Brilliant Blue G-250). A protein band at the correct molecular weight (90–95 kDa) was excised, destained with repeated incubation in 200 mM ammonium bicarbonate, 40% [v/v] acetonitrile. Gel pieces were dried with three washes in 100% acetonitrile and then trypsinised (Trypsin resuspended in 100 mM ammonium bicarbonate, 5% [v/v] acetonitrile) overnight at 37°C. Peptides were extracted from the gel pieces by incubation in 50% [v/v] acetonitrile, 0.1% [v/v] formic acid, peptides were desiccated and resuspended in 3% [v/v] acetonitrile, 0.1% [v/v] formic acid, 20 mM citric acid; pH 2.7. For each analysis, 10% of the peptide sample was loaded onto a nanoACQUITY UPLC Symmetry C18 Trap (5 µm, 180 µm×20 mm) and flow was set to 15 µl/min of 3% [v/v] acetonitrile, 0.1% [v/v] formic acid and 20 mM citric acid for 5 minutes. Analytical separation of the peptides was performed using nanoACQUITY UPLC BEH C18 Column (1.7 µm, 75 µm×250 mm). Briefly, peptides were separated over a 91 minutes solvent gradient from 3% [v/v] acetonitrile, 0.1% [v/v] formic acid to 40% [v/v] acetonitrile, 0.1% [v/v] formic acid on-line to a LTQ Orbitrap Velos (Thermo). Data was acquired using an information dependant acquisition (IDA) method where, for each cycle one full MS scan of *m*/*z* 300–1700 was acquired in the Orbitrap at a resolution of 60,000 at *m*/*z* 400 with an AGC target of 10^6^. Each full scan was followed by the selection of the 20 most intense ions, CID and MS/MS analysis was performed in the LTQ. Selected ions were excluded from further analysis for 60 seconds. Ions with an unassigned charge or a charge of +1 were rejected.

Selected reaction monitoring (SRM) analysis of STAT5A phosphotyrosine 682/683 was performed; the liquid chromatography conditions were as described above. For each cycle, one full MS scan was acquired in the LTQ. Each full scan was followed by MS/MS analysis of parent ions selected in the LTQ with an m/z 477.77 (unphosphorylated Y682 peptide YYTPVLAK), 517.75 (singly phosphorylated) and 557.73 (doubly phosphorylated). Product ions of m/z 216.04, 527.36, 791.47 and 871.43 were scanned for.

SRM analysis was also performed using a LC Packings Ultimate liquid chromatography system and 4000 QTrap (AB Sciex). Typically 30% of the peptide sample was loaded onto a C_18_ trap column using 2% [v/v] acetonitrile in 0.1% [v/v] formic acid (Buffer A) at a flow rate of 30 µL/min and washed for 4 minutes to remove salts. After desalting the flow was reduced to 320 nL/min and diverted to a 15 cm×75 µm i.d. PepMap, C18, 3 µm column. Gradient conditions; the percent of buffer B (80% [v/v] acetonitrile, 0.1% [v/v] formic acid) was increased from 8% to 40% over 21 minutes and then increased to 60% over the following 10 minutes. The flow was held with 60% buffer B content for 2 minutes before being reduced to 8% for equilibration. Separated peptides were eluted from the analytical column directly into the 4000 Q TRAP which was instructed to scan for the parent and product ions described above.

IDA data was analysed using Mascot (Matrix Sciences) the parameters were; Uniprot database, taxonomy *Mus Musculus* or *Homo Sapiens*, trypsin with up to 1 missed cleavage allowed, variable modification were oxidised methionine, phosphorylated serine, threonine and tyrosine and the peptide tolerance of 0.025 Da and 0.03 Da for MS/MS tolerance.

### Determination of Imatinib IC50-values in Human CML Cell Lines

K562 and LAMA-84 cells were lentivirally transduced with control or isoform-specific human STAT5 shRNAs and plated 4 days after transduction in 24-well plates at 1.5×10^4^ cells/mL in the presence of increasing concentrations of imatinib mesylate (0, 0.025, 0.05, 0.1, 0.15, 0.20, 0.25 µM IM). The number of viable cells was determined 96 hours later by Trypan-blue dye exclusion. The number of viable cells was plotted against the concentration of imatinib mesylate and the concentration which reduces this number by 50% of controls was calculated.

## Results

### TonB Cells and Generation of Isoform-specific Anti-STAT5 shRNAs

**Figure 1:**
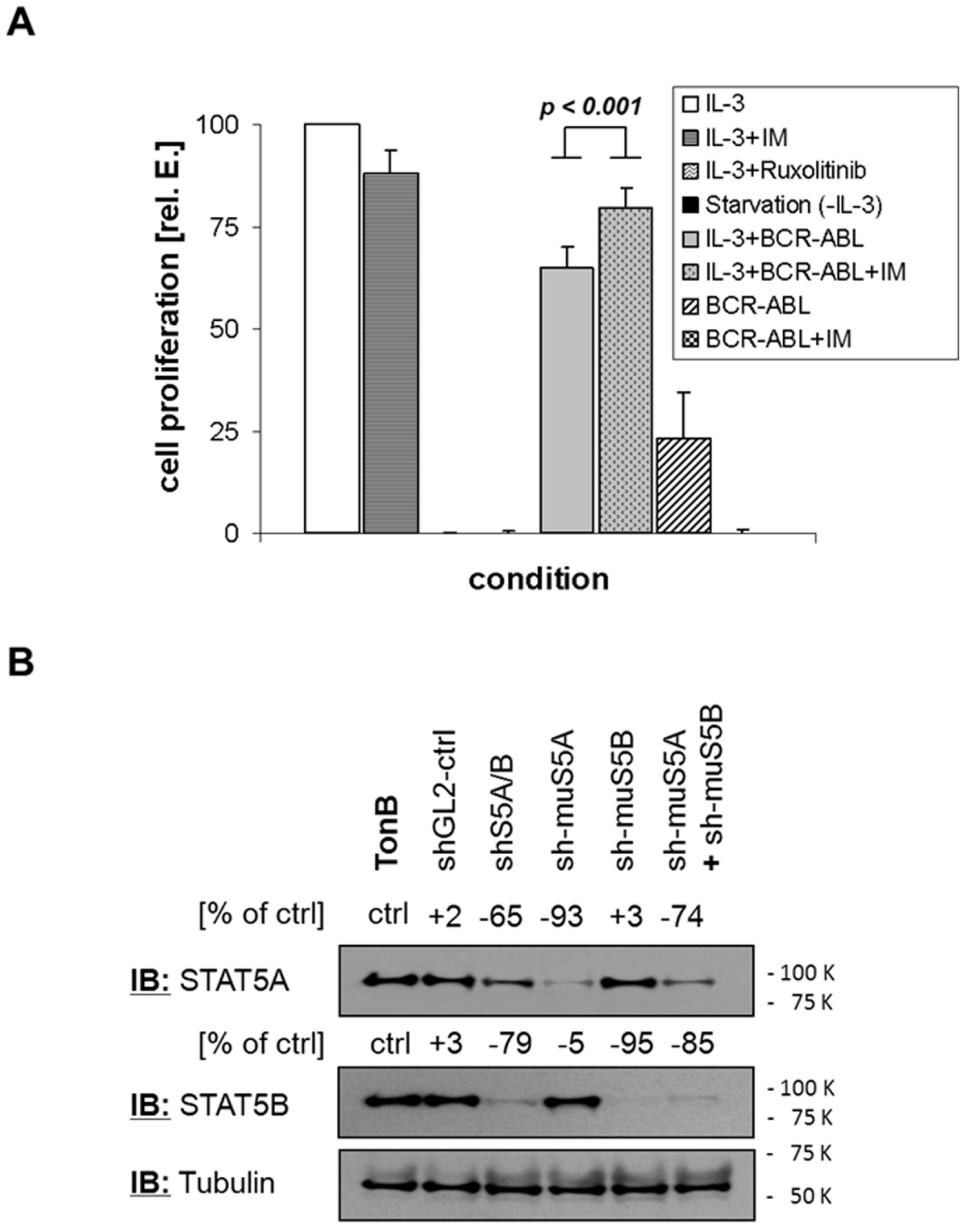
TonB cells and isoform-specific anti-STAT5 shRNAs. (A) TonB cells were cultured in IL-3 (IL-3), IL-3 plus 1 µM imatinib mesylate (IL-3+IM) IL-3+ Ruxolitinib, without IL-3 (Starvation), with IL-3 plus doxycycline (IL-3+BCR-ABL), with IL-3 and doxycycline and imatinib mesylate (IL-3+BCR-ABL+IM), with doxycycline (BCR-ABL) and with doxycycline plus 1 µM imatinib mesylate (BCR-ABL+IM) for 72 hours before viable cells were counted by Trypan-blue dye exclusion. Results are shown as a percentage of control (TonB cells in the presence of IL-3). (B) TonB cells were lentivirally transduced with shRNAs targeting control (shGL2), STAT5A/B (shS5A/B, targeting both isoforms), murine STAT5A (sh-muS5A), murine STAT5B (sh-muS5B), and a mixture of both. Four days after transduction protein expression of both STAT5 isoforms and Tubulin was analyzed by western blotting. The numbers indicate changes in protein expression in % as determined by densitometry.

In the first instance we characterized a system for the study of STAT5 activation by IL-3 or BCR-ABL or both using TonB cells, a murine IL-3-dependent pro B-cell line with doxycycline inducible BCR-ABL-expression [27]. Induction of BCR-ABL protein-expression reaches up to 87% of that seen in the human K562 CML cell line in a dose- and time-dependent manner (Figure S1). The TonB model allows direct comparison between IL-3 and BCR-ABL signalling at identical expression levels of STAT5-isoforms and/or relevant cofactors. It was determined that optimal proliferation of these cells is observed in the presence of IL-3 whereas BCR-ABL-mediated proliferation is only about 25% of this IL-3 effect. IL-3 plus BCR-ABL achieved about 70% of the optimal levels of proliferation observed (Figure 1A). Interestingly, inhibition of BCR-ABL tyrosine kinase activity by Imatinib (IM) further improved TonB cell proliferation in the presence of IL-3 and BCR-ABL (Figure 1A). These data demonstrate some kind of interference of BCR-ABL- with IL-3R- signalling in TonB cells most likely by competition and differential impact on common signalling components.

We next designed isoform-specific anti-STAT5 shRNAs to discriminate between the actions of the A- and B-isoforms. The most effective shRNA was selected out of 5 as previously described [24] and western blot analysis revealed isoform-specific reduction of STAT5A and STAT5B expression (>90% with very low cross-reactivity, ≤5%, by the respective shRNAs) (Figure 1B).

### Isoform-specific STAT5 Loss-of-function Phenotypes in TonB Cells

**Figure 2:**
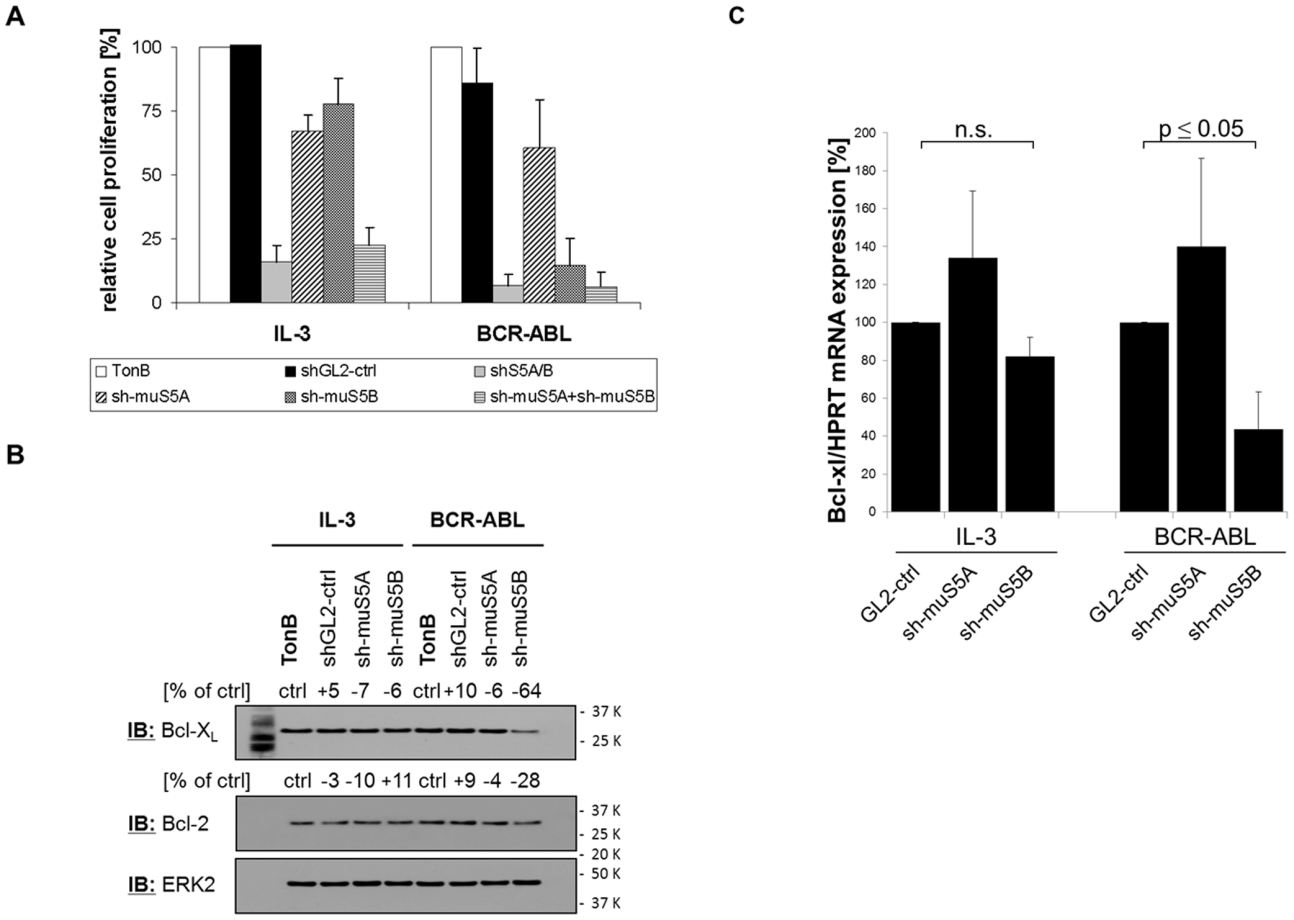
Isoform-specific STAT5 loss-of-function phenotypes in TonB cells. (A) TonB cells were lentivirally transduced with shRNAs targeting control (shGL2), STAT5A/B (shS5A/B), murine STAT5A (sh-muS5A), murine STAT5B (sh-muS5B), and a mixture of both and cultured in the presence of IL-3. Four days after transduction cells were either cultured with IL-3 or switched to BCR-ABL, and the number of viable cells was determined after additional four days by Trypan-blue dye exclusion. The proliferation of non-transduced cells under IL-3 and BCR-ABL was set 100%, respectively. The data represent mean of ten independent experiments. (B) TonB cells transduced with isoform-specific anti-STAT5 shRNAs were cultured four days in the presence of IL-3 or BCR-ABL. Protein expression of BCL-X_L_ and BCL-2 was analysed by western blotting. Expression of ERK2 served as loading control. The numbers indicate changes in protein expression in % as determined by densitometry. (C) TonB cells lentivirally transduced with isoform specific STAT5 shRNAs were cultured in the presence of IL-3 or doxycycline as described in (B). mRNA expression was quantified by qRT-PCR. BCL-X_L_ expression was normalized to HPRT mRNA-expression, and GL2-ctrl was set as 100%. The data represent mean ± SE of four independent experiments.

TonB cells grown in the presence of IL-3 or maintained by BCR-ABL action were subjected to isoform-specific anti-STAT5 shRNA treatment. We observed that anti-STAT5A and anti-STAT5B shRNAs reduced the number of viable cells by 33% and 23%, respectively, (IL-3 as stimulus) and by 40% and 85%, respectively, (BCR-ABL) compared to control shRNAs (Figure 2A). This decrease in number of viable cells is due to apoptosis as shown by an increase in Sub-G1-fraction (Figure S2). Protein expression of BCL-X_L_, a known target of STAT5, is reduced in BCR-ABL- but not in IL-3-supplemented cultures with reduced STAT5B (sh-muS5B) but not STAT5A (sh-muS5A) expression (Figure 2B). In contrast, BCL-2 expression is only slightly affected by reduction of STAT5 expression under both culture conditions. Similarly, BCL-X_L_ mRNA expression is significantly diminished only in BCR-ABL-expressing but not IL-3 supplemented cultures of TonB cells with reduced STAT5B expression as compared to controls (sh-muS5B 43.7% +/−19.4%, p = 0.03; Figure 2C). These data demonstrate different STAT5A activity in the presence of IL-3 and BCR-ABL and suggest specific functions of STAT5B in TonB cells in the presence of BCR-ABL.

### Isoform-specific STAT5 Gain-of-function Phenotypes in TonB Cells

**Figure 3:**
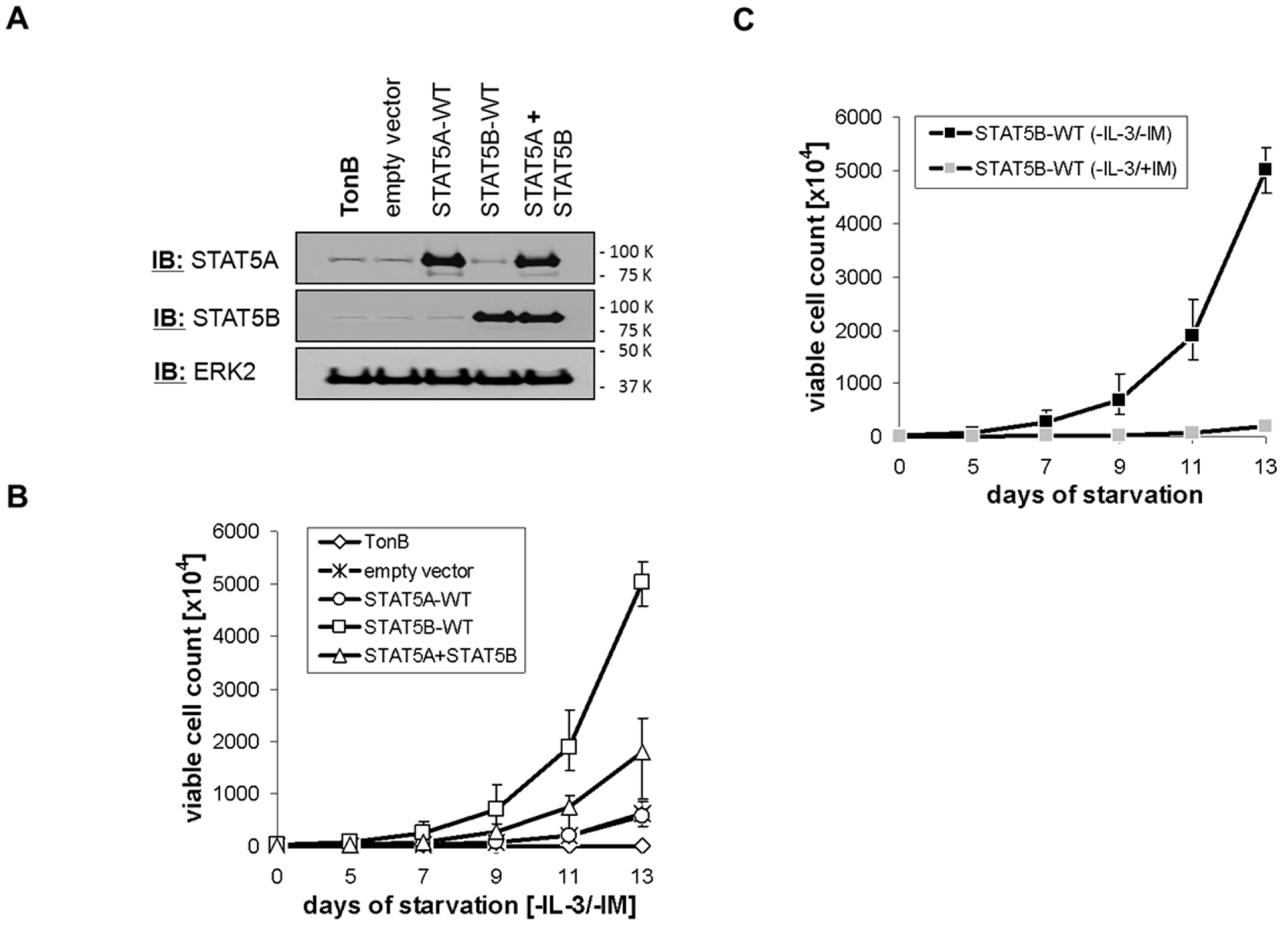
Over-expression of STAT5 isoforms in TonB cells. (A) Expression of STAT5 isoforms was analysed by western blotting of TonB cells transduced with empty vector or murine cDNAs encoding STAT5A or STAT5B four days before. (B) TonB cells transduced with empty vector or STAT5 isoforms as shown in (A) were starved by removal of IL-3, and the number of viable cells was determined by Trypan-blue dye exclusion over time. The data represent mean of five independent experiments. (C) TonB cells over-expressing STAT5B as shown in (A) and (B) were cultured in the presence (+IM) or absence (−IM) of 1.5 µM imatinib mesylate and the number of viable cells was plotted over time. The data represent mean of 3 independent experiments. Due to the scale standard deviation for the –IL-3/+IM condition is not visible.

To investigate these differential effects further, TonB cells were transduced to over-express STAT5A or STAT5B. Increased expression was confirmed by western blotting (Figure 3A). Four days after transduction IL-3 was removed and the number of viable cells was determined over time. Over-expression of STAT5B increased cell survival and proliferation much more efficiently than that of STAT5A with intermediate cell proliferation upon over-expression of both STAT5A and STAT5B (Figure 3B). Furthermore, tyrosine phosphorylation of STAT5B is detectable in TonB cells over-expressing STAT5B in the absence of IL-3 and doxycycline (Figure S3). Interestingly, STAT5B-dependent proliferation was reduced about 20-fold in the presence of imatinib although cells were cultured without doxycycline (Figure 3C). Parental BaF3 cells were not transduced to factor-independent growth by over-expression of either STAT5A or STAT5B under these conditions (data not shown). These data suggest a low basal BCR-ABL expression level in non-induced TonB cells and enhancement of BCR-ABL signalling by over-expression of STAT5B. In addition, they correlate with the functional role of STAT5B in maintenance of anti-apoptotic BCL-X_L_ levels in the presence of BCR-ABL (Figure 2B and C).

### STAT5 Phosphorylation by IL-3 Receptor/JAK2 and BCR-ABL Signalling

**Figure 4:**
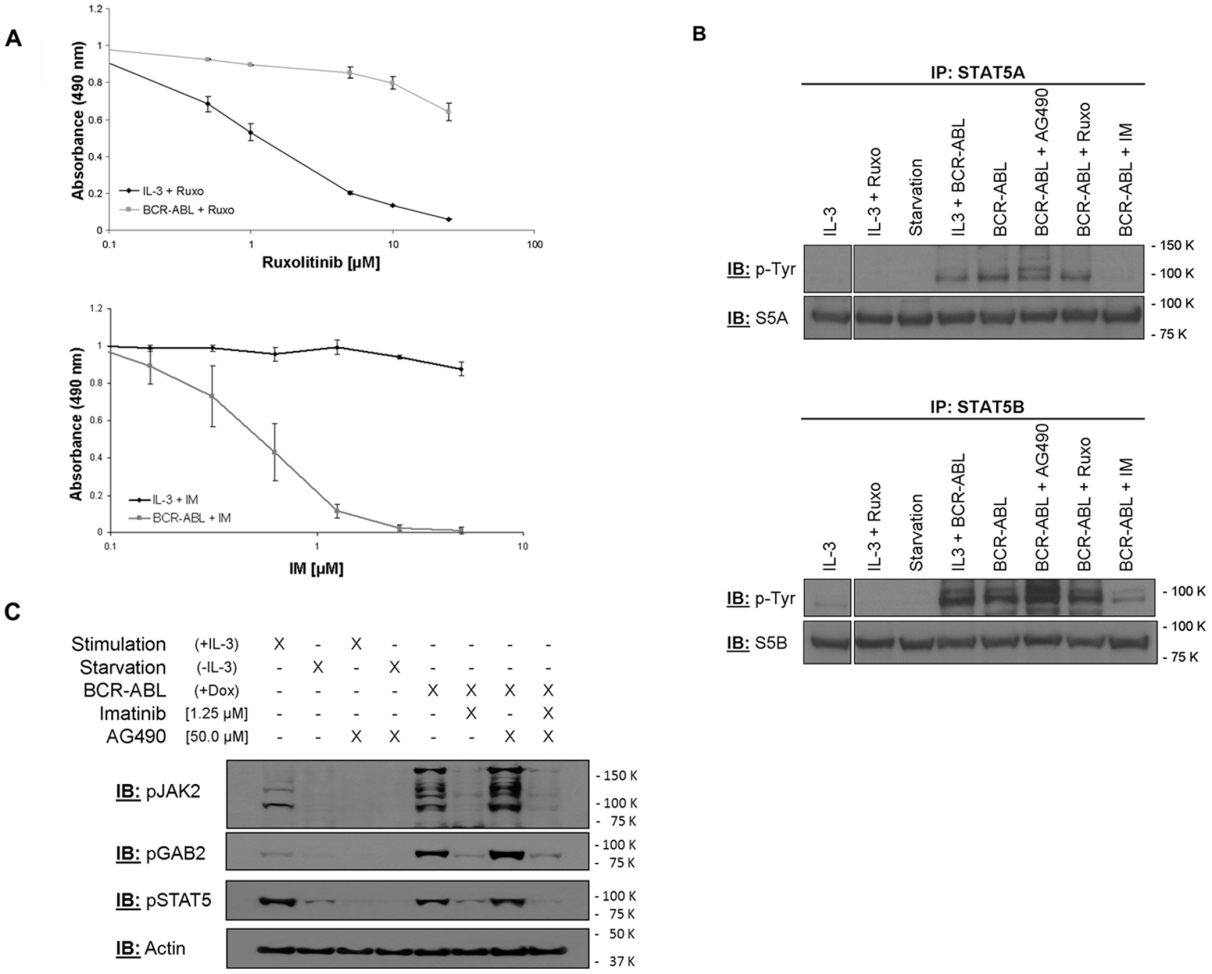
STAT5A and STAT5B phosphorylation by IL-3 receptor and BCR-ABL signalling. (A) TonB cells cultured with IL-3 or BCR-ABL-expressing cells were treated with different concentrations of Ruxolitinib (0.5 µM –25 µM) or Imatinib (0.15 µM –5 µM). Cells were incubated for 48 hours and proliferation was measured using MTS. Data represent mean ± SD of three independent experiments. (B) TonB cells were cultured in the presence of IL-3, IL-3+ doxycycline to induce BCR-ABL-expression or doxycycline alone. Aliquots were treated with Ruxolitinib (5 µM), starved by withdrawal of IL-3 (Starvation), AG490 (50 µM) or 1 µM imatinib mesylate for 24 hours. Cells were lysed and STAT5A (upper panel) and STAT5B (lower panel) were immunoprecipitated with isoform-specific anti-STAT5 antibodies. Tyrosine phosphorylation and the amount of STAT5 (loading control) were analyzed by western blotting. (C) TonB cells were cultured either in the presence of IL-3 (+IL-3) or BCR-ABL (+Dox). After starvation (20 hours in the absence of IL-3) cells were replated in the presence or absence of IL-3 and the JAK2 kinase inhibitor AG490 at final concentrations of 50 µM. BCR-ABL-expressing cells were split and supplied with either imatinib at final concentrations of 1.5 µM and/or AG490 at final concentrations of 50 µM. Twenty hours later equal numbers of viable cells were directly lysed in SDS-boiling buffer and subjected to SDS-PAGE. The phosphorylation levels of JAK2, GAB2, and STAT5 were analyzed by western blotting using antibodies recognizing the following phospho-epitops: Tyr1007/1008 (JAK2); Tyr452 (GAB2); and Y694 (STAT5). Expression of Actin served as loading control. One out of three representative experiments is shown.

We next attempted to analyze STAT5 tyrosine phosphorylation by IL-3 and BCR-ABL respectively, using pharmacological JAK2 and BCR-ABL inhibitors. Since JAK2 constitutively binds to the IL-3 receptor β-chain we first analyzed the role of JAK2 kinase activity on BCR-ABL-induced STAT5 tyrosine phosphorylation in the presence or absence of ruxolitinib, a specific JAK2/JAK1-inhibitor (for review see [28]). Ruxolitinib inhibits IL-3 induced cell proliferation more efficiently than that driven by BCR-ABL (Figure 4A). In addition, IL-3 induced STAT5 tyrosine phosphorylation is inhibited by ruxolotinib (Figure 4B). In contrast, STAT5 tyrosine phosphorylation in the presence of BCR-ABL is only inhibited by imatinib but not by ruxolitinib. These data demonstrate tyrosine phosphorylation of STAT5 by BCR-ABL even if JAK2 is inhibited to prevent cell proliferation.

To analyze the function of additional cytoplasmic tyrosine kinases beyond JAK2 we used the tyrosine kinase inhibitor AG490 which completely inhibited IL-3 induced tyrosine phosphorylation of JAK2, STAT5 and GAB2 (Figure 4C). In contrast, BCR-ABL mediated tyrosine phosphorylation of all three molecules is again only inhibited by imatinib, but remains unaffected by AG490 at a dose no tyrosine phosphorylation is detectable under IL-3 treatment. Taken together, these data suggest tyrosine phosphorylation of JAK2, STAT5 and GAB2 by BCR-ABL even in the absence of JAK2 kinase activity. However, JAK2 itself can be tyrosine phosphorylated by BCR-ABL in the absence of cytokine stimulation and may thereby propagate BCR-ABL signals. Thus oncogenic signalling through JAK/STAT pathway is different to that mediated by cytokines in this model.

### Hetero- and Homodi-(oligo)merization of STAT5A and STAT5B

**Figure 5:**
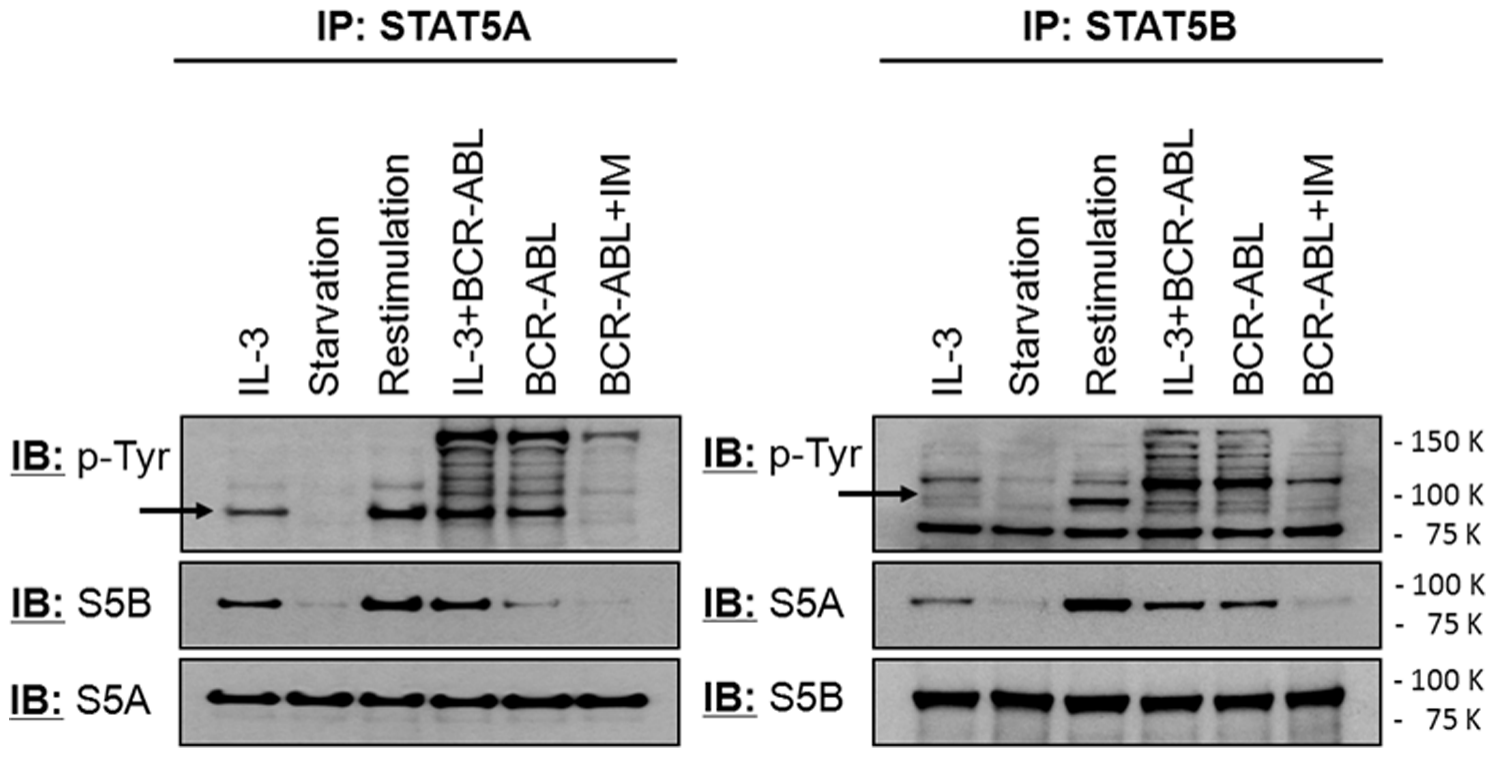
Heterodi-(oligo)merization of STAT5A and STAT5B in TonB cells. TonB cells were cultured in the presence of IL-3, BCR-ABL, and IL-3 plus BCR-ABL. Aliquots were starved overnight by withdrawal of IL-3 (Starvation) and re-stimulated with IL-3 for 30 minutes (Restimulation) or were treated with 1 µM imatinib mesylate (BCR-ABL+IM). Cells were lysed and STAT5A (left) and STAT5B (right) were immunoprecipitated with isoform-specific anti-STAT5 antibodies. Overall tyrosine phosphorylation of precipitated proteins (upper panel), co-precipitation of the other STAT5 isoform (middle panel), and the amount of precipitated STAT5 as a loading control (lower panel) were analyzed by western blotting.

There is data on STAT molecules as monomers, pre-formed or active dimers [2] and the interplay between STAT5A and STAT5B, we therefore next considered physical association between these two proteins. STAT5A and STAT5B were immunoprecipitated from whole cell lysates of native TonB bulk cultures under different conditions using isoform-specific antibodies. When BCR-ABL was active, imatinib blocked STAT5A:STAT5B heterodimerization as indicated by reduced co-immunoprecipitation of STAT5A and STAT5B (Figure 5). Thus this process is clearly BCR-ABL tyrosine kinase sensitive. This may be linked to tyrosine phosphorylation and indeed tyrosine phosphorylation of STAT5A and STAT5B is detectable if either cells are cultured in IL-3 and/or BCR-ABL tyrosine kinase activity is present. However, tyrosine phosphorylation of STAT5B appeared to be relatively weaker compared to STAT5A under steady state conditions (Figure 5, right panel). Co-immunoprecipitation of STAT5A and STAT5B was also observed in the presence but not in the absence of IL-3 and again correlated to STAT5 tyrosine phosphorylation. The situation was more complex in the presence of BCR-ABL and IL-3. STAT5A immunoprecipitates in BCR-ABL-expressing cells have about two third less STAT5B present compared to BCR-ABL plus IL-3-treated cells, although tyrosine phosphorylation levels were similar (Figure 5, left panel). In the complementary experiment with STAT5B immunoprecipitates, there was at most only a marginal increase in co-immunoprecipitated STAT5A detectable in the presence of IL-3 and BCR-ABL as compared to BCR-ABL-only cultures (Figure 5, right panel). Finally, BCR-ABL kinase activity induced co-immunoprecipitation of several tyrosine phosphorylated proteins with STAT5A and STAT5B, respectively, and this was independent of the presence of IL-3. IL-3 alone did not achieve such an effect. Thus there is a clear demonstration that phosphotyrosine-dependent STAT5A:STAT5B-heterodimerization or -oligomerization is affected by BCR-ABL.

One potential inference from the above data is that BCR-ABL alters the formation of homo- or heteromeric STAT5 complexes by favouring tyrosine phosphorylated STAT5 in monomeric or inactive homomeric conformation and/or heterologous complexes with other signalling molecules. We therefore analyzed homodi- or oligomerization of STAT5 isoforms using epitope-tagged STAT5A and STAT5B variants and found constitutive and phosphotyrosine-independent homodi- or oligomerization of both STAT5A and STAT5B (Figure S4A). These non-functional homodi- or oligomers of both STAT5A and STAT5B are mostly in the cytoplasm and only about 20% in the nucleus independent of the presence or absence of IL-3 (Figure S4B).

### Intracellular Localization of STAT5A in the Presence of BCR-ABL

**Figure 6:**
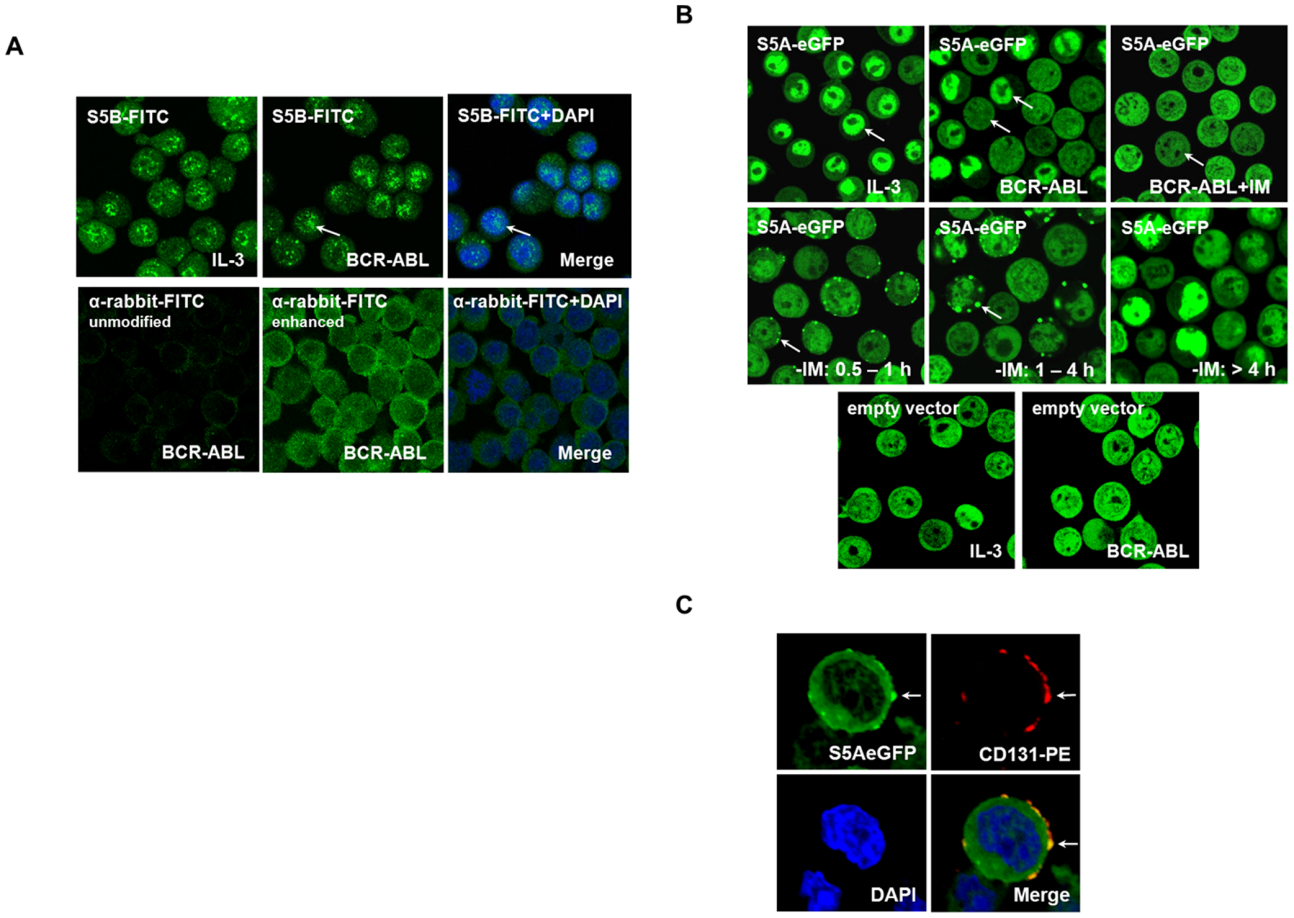
Intracellular localization of STAT5 isoforms. (A) TonB cells cultured in the presence of IL-3 or doxycycline were spun down on glass slides, fixed by ice-cold methanol and stained sequentially with anti-STAT5B, anti-rabbit-FITC and DAPI. Intracellular localization was analyzed using confocal microscopy. Nuclear localization was confirmed by a DNA-specific DAPI counterstain. The lower panel shows antibody (with and without enhancement) and DAPI control. (B) TonB cells expressing STAT5A-eGFP were cultured in the presence of IL-3, BCR-ABL or BCR-ABL plus imatinib mesylate (BCR-ABL+IM) and live-cell analysis was performed by confocal microscopy (upper panel). The middle panel shows STAT5A-eGFP in BCR-ABL-expressing TonB cells after removal of imatinib mesylate (−IM) for the indicated periods of time. The lower panel shows empty vector control (eGFP). (C) TonB-STAT5A-eGFP cells treated as in Figure 6B, middle panel, were fixed 45 minutes after imatinib-removal, stained with anti-CD131-PE antibody (IL-3/IL-5/GM-CSF-receptor common β-chain) and DAPI before analysis by confocal microscopy.

To investigate subcellular localization of STAT5 isoforms in the presence and absence of IL-3 and BCR-ABL TonB cells were stained with isoform-specific anti-STAT5 antibodies and analyzed by confocal microscopy. STAT5B is mainly found within the nucleus of TonB cells in the presence of either IL-3 or BCR-ABL (Figure 6A). In contrast to STAT5B we could not visualize intracellular STAT5A distribution by this approach. We therefore expressed a STAT5A-eGFP fusion protein in TonB cells which accumulates in the nucleus in almost all cells in the presence of IL-3. In contrast, only approximately 50% of cells show nuclear localisation of STAT5A-eGFP in the presence of BCR-ABL (Figure 6B, upper panel). When BCR-ABL tyrosine kinase activity is blocked by imatinib, no nuclear accumulation of STAT5A-eGFP is detectable. Release of tyrosine kinase inhibition by washing out imatinib induced rapid translocation of STAT5A-eGFP to the cell membrane within minutes in a fraction of cells (Figure 6B, middle). Here STAT5A-eGFP co-localizes with the IL-3 receptor β-chain (Figure 6C) before it eventually accumulates within the nucleus. These data demonstrate specific effects of BCR-ABL on intracellular localization of STAT5A in line with defective activation by this oncogene. We could not perform corresponding experiments with a STAT5B-RFP transgene since over-expression of STAT5B-RFP is transforming in TonB cells.

### Aberrant Tyrosine Phosphorylation of STAT5A in the Presence of BCR-ABL

**Figure 7:**
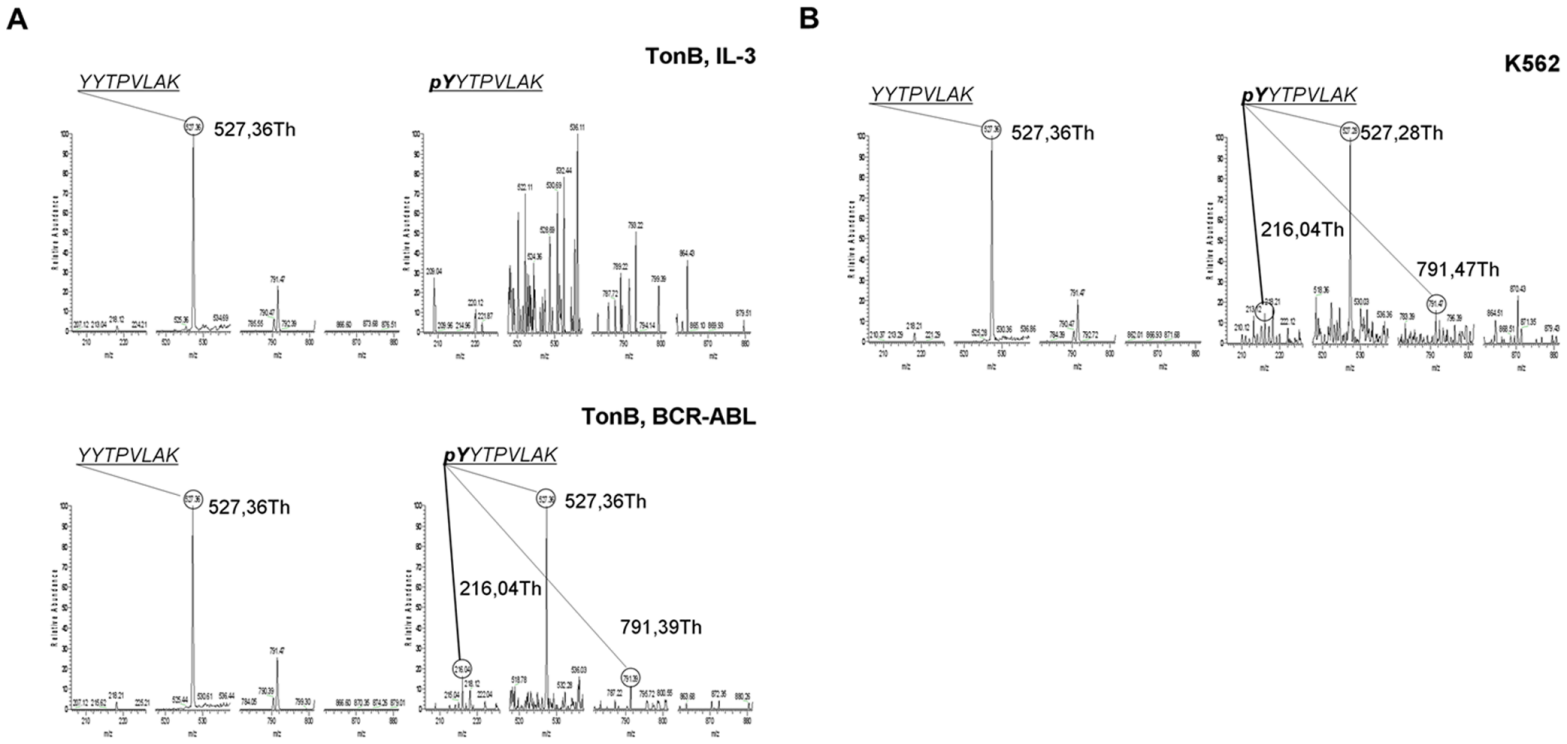
Mass spectrometry-based analysis of aberrant STAT5 tyrosine phosphorylation. For each part of the figure there are 2 panels, the left hand panel the parent ion transition is 477.77Th (unphosphorylated STAT5A) and the right hand panel the parent ion transition is 517.75Th (phosphorylated STAT5A). (A) STAT5 was isoform-specific immunoprecipitated from TonB cells in the presence of IL-3 or BCR-ABL. A peptide (YYTPVLAK) in STAT5A was initially found to be phosphorylated on Y682 in the presence of BCR-ABL but not in the presence of IL-3. Ions with the appropriate parent mass/charge ratios were selected for tandem mass spectrometric analysis. Product ions 527.3552Th (identifying the peptide), 871.4325Th (indicative for pY683), 791.4662Th (indicative for pY682), and 216.04Th (indicative for phosphorylated tyrosine) are labelled. A list of all product ions is shown in Figure S5. (B) STAT5A was precipitated from K562 cells and analyzed as described above.

The above data show that BCR-ABL and IL-3 differentially affect STAT5A and STAT5B. This could be achieved via different tyrosine phosphorylation by the BCR-ABL tyrosine kinase activity (or its downstream activation of other protein kinases). A potential novel phosphorylation site on STAT5A or STAT5B was therefore investigated using a mass spectrometry approach. From an initial discovery based proteomic analysis it was determined that either STAT5A Y682 or Y683 was phosphorylated. Using immunoprecipitated STAT5A from TonB cells treated with IL-3 or doxycycline, selected reaction monitoring (SRM) analysis was used to verify the site of phosphorylation from the peptide YYTPVLAK. As shown in Figure S5 the site of phosphorylation can be determined due to the presence or absence of the product ions at 791.4662Th or 871.4325Th from the parent ion 517.77Th (the doubly charged, phosphorylated YYTPVLAK). Also the immonium ion generated at 216.04Th by decomposition of phosphotyrosine present in the peptide is diagnostic for tyrosine phosphorylation substantiating the likely tyrosine phosphorylation. Transfected STAT5A was immunoprecipitated from TonB cells, and SRM was performed, this demonstrated that Y682 was phosphorylated (Figure 7A). In Figure 7A (lower right panel) ions at 791.4Th and 216.04Th were detected, indicative of a phosphorylation event on Y682 in the presence of BCR-ABL. There was no signal seen at 871.4325Th (diagnostic for phosphorylation on the second tyrosine residue; Y683), thus further indicating correct assignment of the site of phosphorylation being Y682. Interestingly in the top right panel (TonB cells in the presence of IL-3) the phosphorylated Y682 site was not observed. A final validation of the site of phosphorylation was seen when Y682 and/or Y683 were mutated to phenylalanine (Figure S6A–C). Although a product ion was seen in both single mutants at 492.16Th (phosphorylated Y at either 682 or 683) no product ion was detected at 527.3552Th for the Y682F, thus the 492.16Th ion in this case could be a contaminant. However when the Y683F mutant was analyzed (Figure S6B) both ions at 527.3552Th and 492.16Th were seen (Figure S7).

It was also attempted to identify this phosphorylation event in human K562 cells (Figure 7B) and in primary CML cells (Figure S6D). In both cases a signal of the product ion at 791.47Th from the appropriate parent ion was detected, indicating that the pY682 event is present in these samples.

Phosphorylation of the well characterized tyrosine residues Y694 in STAT5A and Y699 in STAT5B was observed in the presence of both IL-3 and BCR-ABL. However, analysis of STAT5B led to no identification of any phosphorylation from the homologous site on STAT5B (Y682).

Finally, we over-expressed STAT5A Y682F, Y683F and Y682/683F mutants in TonB cells and analyzed cell proliferation as well as intracellular localization and complex formation of STAT5A mutants. So far, we could only detect reduced complex formation with heterologous proteins in particular for STAT5A Y682/683F double mutants (Figure S8) but no specific effects on cell proliferation or cellular localisation of STAT5A (data not shown).

### Function of STAT5 Isoforms in Human Cells

**Figure 8:**
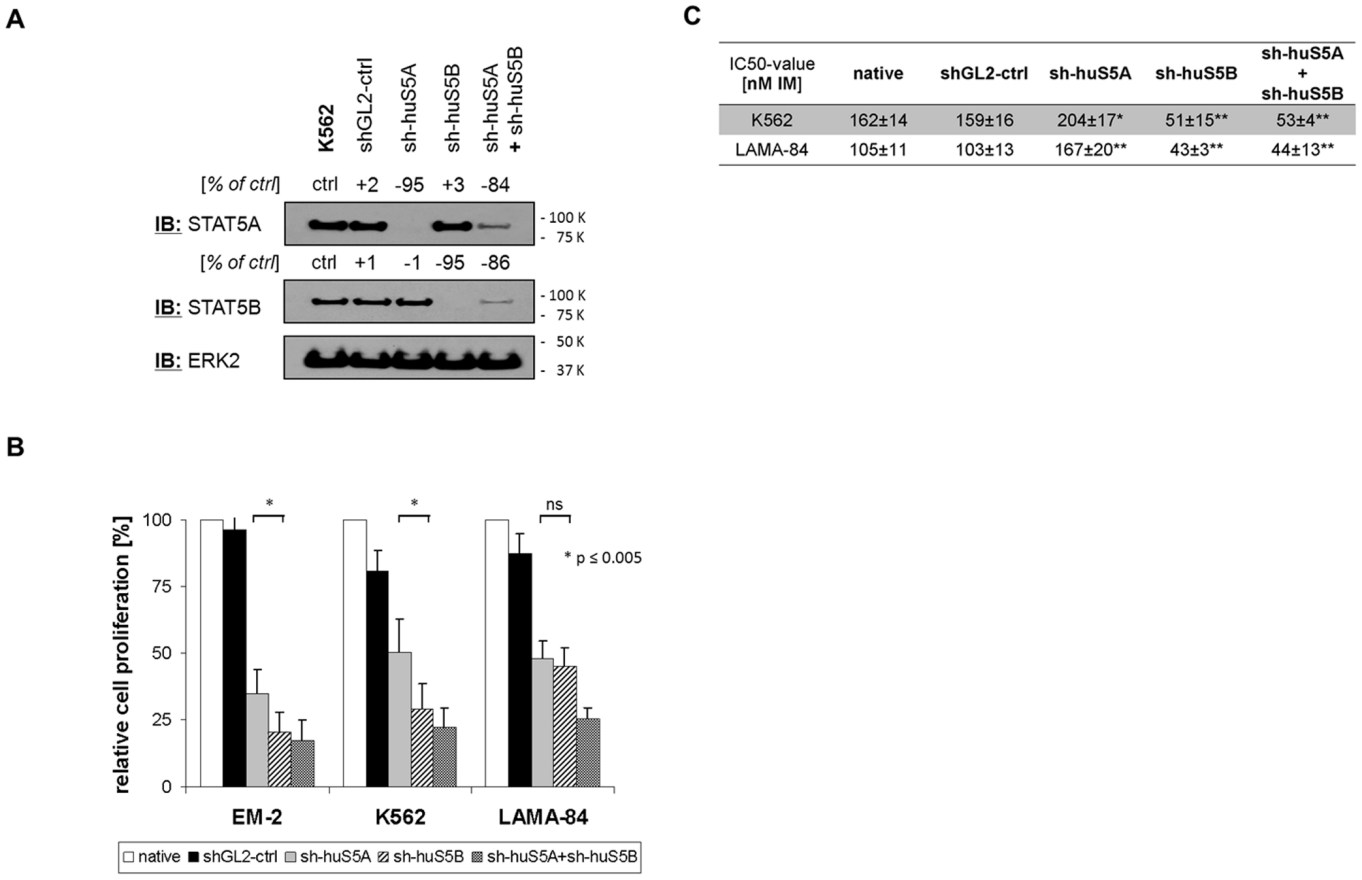
Isoform-specific inhibition of STAT5 in human cell lines. (A) K562 cells were lentivirally transduced with shRNAs targeting control (shGL2), human STAT5A (sh-huS5A), human STAT5B (sh-huS5B), and a mixture of both. Four days after transduction protein expression of both STAT5 isoforms and ERK2 was analyzed by western blotting. The numbers indicate changes in protein expression in % as determined by densitometry. (B) The human BCR-ABL-positive cell lines EM-2, K562 and LAMA-84 were lentivirally transduced with control (shGL2), isoform-specific (sh-huS5A, sh-huS5B), and a mixture of both isoform-specific shRNAs. Four days after transduction, equal numbers of cells were plated and the number of viable cells was determined after additional four days by Trypan-blue dye exclusion. The number of viable non-transduced cells (native) was set 100%. The data represent mean of four independent experiments. (C) Impact of isoform-specific STAT5 shRNAs on imatinib mesylate (IM) response as determined by IC50. STAT5 shRNAs were compared to GL2 controls with * p<0.05 and ** p<0.01.

To study the different functions of STAT5A and STAT5B in human cells shRNAs targeting human STAT5A and STAT5B were generated as described above. Again isoform-specific shRNAs specifically inhibit protein expression of human STAT5 isoforms with low cross-reactivity (Figure 8A). Upon lentiviral transduction both anti-STAT5A and anti-STAT5B shRNAs inhibit cell proliferation and survival of human BCR-ABL-positive cell lines to a similar extent (Lama-84) or slightly stronger in the presence of anti-STAT5B shRNA (K562, EM-2) (Figure 8B). In addition, depletion of STAT5B but not STAT5A sensitizes K562 and Lama-84 cells to imatinib with a 2.4- to 3.2-fold reduction of IC50 (Figure 8C). In contrast, anti-STAT5A RNAi slightly increases the IC50 in both cell lines studied, and the combination of both shRNAs was not superior to anti-STAT5B shRNA alone. In EM-2 cells anti-STAT5A and anti-STAT5B shRNAs alone reduced viability to more than 40% so IC50 could not reliably be determined. In contrast, depletion of STAT5A or STAT5B did not differentially affect cytokine-stimulated colony formation of chronic phase CML CD34+ cells (Figure S9). However, these data demonstrate specific biological effects of STAT5B for BCR-ABL-driven proliferation in human cell lines in line with biochemical study described above.

## Discussion

Here we have considered differential effects of IL-3 and BCR-ABL on STAT5 isoforms. Our data demonstrate differences between IL-3- and BCR-ABL-mediated STAT5-activation with STAT5A activation by BCR-ABL being partially deficient. This defective activation of STAT5A may explain the only partial rescue of BCR-ABL-dependent proliferation by the addition of IL-3 which can be further enhanced by inhibition of BCR-ABL tyrosine kinase activity (Figure 1A). Accordingly, depletion of STAT5B has more impact on BCR-ABL-expressing than on IL-3-supplemented TonB cells indicating that the remaining STAT5A function can only partially rescue loss of STAT5B in the presence of BCR-ABL (Figure 2A). Similarly, BCL-X_L_ expression is only affected in BCR-ABL but not in IL-3 cultures by depletion of STAT5B (Figure 2B and C). Finally, over-expression of STAT5B but not STAT5A can expand TonB cells in the absence of IL-3, but this transformation still depends most likely on low level BCR-ABL tyrosine kinase activity (Figure 3B and C and Figure S3). These and the related data on reduced IC50-values for imatinib upon depletion of STAT5B but not STAT5A in human cell lines (Figure 8C) indicate a crucial role of STAT5B/BCR-ABL interactions for BCR-ABL-induced cell proliferation.

The mechanism of differential STAT5 activation by IL-3R/JAK2 and BCR-ABL is not yet known. BCR-ABL can induce tyrosine phosphorylation of STAT5, JAK2 as well as GAB2 even in the absence of both cytokine stimulation and JAK2 kinase activity (in AG490 supplemented cultures). Since only imatinib but neither ruxolitinib nor AG490 inhibits BCR-ABL-dependent tyrosine phosphorylation of JAK2 (Figures 4A and B) JAK2 that constitutively binds to the IL-3R β-chain [29], [30] is phosphorylated either directly by BCR-ABL or by another kinase not inhibited by AG490. Similarly, STAT5 (and GAB2) may be phosphorylated either directly by BCR-ABL and/or by JAK2 (e.g. upon activation by BCR-ABL) and/or by other kinases such as HCK [31]. Our observations are in line with and expand recently reported data that BCR-ABL uncouples JAK/STAT signalling and may directly induce STAT5 tyrosine phosphorylation [32].

Different mechanisms of phosphorylation by IL-3R/JAK2 and BCR-ABL may affect STAT5 conformation and function since heterodimerization of STAT5A with STAT5B is more efficient upon stimulation by IL-3 compared to BCR-ABL stimulation. STAT5 molecules dimerize in parallel (active) and anti-parallel (inactive, constitutive) conformations with either a p-STAT or non p-STAT nuclear entry [2], [33], and STAT activation is linked to monomer dimerization or the transition between these different dimeric conformations [34], [35]. Since co-localization with the IL-3R β-chain even in the absence of IL-3 precedes nuclear localization of STAT5A-eGFP in the presence of BCR-ABL (Figure 6B and C), IL-3R may provide structural support as well as scaffolding function for generation of active STAT5 dimers. In contrast, formation and localization of constitutive STAT5A and STAT5B homodimers or oligomers, at least upon over-expression, are not affected by BCR-ABL (Figure S4). Our data are in line with a model of receptor-linked tyrosine phosphorylation (IL-3R or Flt3-ITD [36]) resulting in formation of active dimers whereas tyrosine phosphorylation by cytoplasmic kinases such as BCR-ABL or src-kinases may favor inactive di- or oligomerization and even heterologous complex formation (Figure 5). The underlying mechanism may involve aberrant binding of pSTAT5 via its own SH2 domain e.g. to phosphorylated v-src [36] or recognition of STAT5 phosphotyrosine residues by SH2- or PTB-domains of other signalling molecules. Both, the canoncical Y694 as well as Y682/Y683 in STAT5A may provide such binding sites since at least simultaneous mutation of both Y682 and Y683 reduces the amount of proteins co-immunoprecipitated with STAT5A (Figure S8).

Several functional differences between STAT5A and STAT5B have been described. For example, STAT5B can induce STAT5A expression but not vice versa in v-ABL expressing cells [37], and differential contribution to stress response has been reported [38]. Furthermore, differences in subcellular localization [39] have been described. Since we could only analyze the localization of endogenous and transgenic STAT5B and STAT5A, respectively, we cannot directly compare their subcellular localization in the context of BCR-ABL. However, our data indicate some cytoplasmic retention of STAT5A but not STAT5B in the presence of BCR-ABL (Figure 6). Interestingly, IL-3 normalizes nuclear accumulation of STAT5A in the presence of BCR-ABL (data not shown) suggesting that IL-3R induced STAT5 activation remains intact. However, the exact role of assembly of IL-3R, STAT5, JAK2, and BCR-ABL for phosphorylation and STAT5 activation and location needs to be further defined.

In addition to structural requirements for STAT5 activation, aberrant phosphorylation of STAT5A at Y682 may impact on the molecular dynamics of STAT5 localization, recruitment into signalling complexes, and formation of functional homo- and heterodimers. We observed tyrosine phosphorylation of STAT5A-Y682 in the presence of BCR-ABL in TonB cells, K562 cells and in a primary CML sample, but did not detect an effect of IL-3 on this site (using mass spectrometry). Our analyses also did not detect the corresponding site in STAT5B. Interestingly, STAT5A and STAT5B differ most from each other at this specific site: instead of L687 (STAT5A) a unique six amino acid insertion (PCEPAT in mouse, PCESAT in human) to the otherwise perfect alignment of both STAT5 isoforms is found in STAT5B. Twin-YY-motifs may serve as intersections for protein-protein interactions after post-translational modification and have been shown to regulate e.g. JAK kinase activity [40]–[44]. Since we only found some impact of Y682 and Y683 on STAT5A complex formation with heterologous molecules (Figure S8), the exact role of differential phosphorylation at Y682 in STAT5A requires further studies. Finally, our MS analyses do not allow quantification of aberrant STAT5A-Y682 phosphorylation by BCR-ABL.

What of the functional role of defective STAT5A activation by BCR-ABL in human cells? Firstly, it may explain the presence of tyrosine phosphorylated STAT5 in the cytoplasm as reported by several groups [36], [38], [45]. Furthermore, loss-of-function of STAT5B reduces the IC50 for imatinib about 3-fold in K562 and LAMA-84 cells, whereas RNAi targeting STAT5A slightly increases it (Figure 8C). These data point to specific STAT5B-BCR-ABL interactions required for proliferation in human cells too. In cytokine-stimulated colony assays of CML CD34+ cells, however, we could not detect isoform-specific effects of anti-STAT5 shRNAs (Figure S9). This may be due to the cytokine stimulation required in these assays which competes with BCR-ABL and may sufficiently stimulate STAT5 isoforms. Furthermore, the data presented suggest a yet unknown potential mechanism of drug resistance in the absence of BCR-ABL kinase mutations: up-regulation of STAT5B expression may allow STAT5 signalling in the presence of reduced BCR-ABL kinase activity (Figure 8C, Figure 3C) corresponding to enhanced resistance against tyrosine kinase inhibitor treatment. These data are in line with earlier reports on up-regulation of STAT5 expression and STAT5 phosphotyrosine-dependent drug effects in kinase inhibitor-resistant and advanced phase CML [46]. Finally, the data provide molecular evidence that STAT5B may represent a specific therapeutic target in BCR-ABL-positive leukemia although the development of STAT5B-specific small molecule inhibitors may be difficult due to the high homology between STAT5A and STAT5B [47]. This may be relevant for clinical conditions with limited efficacy of tyrosine kinase inhibitory therapy such as primary resistant CML and BCR-ABL-positive ALL.

In summary, our data suggest the following model. Receptor linked tyrosine phosphorylation favours the generation of active STAT5 dimers in parallel conformation which translocate into the nucleus [2] whereas cytoplasmic phosphorylation favors the formation of inactive and even heterologous STAT5 complexes retained in the cytoplasm [36]. Upon phopshorylation by BCR-ABL this cytoplasmic retention is more pronounced for STAT5A shifting STAT5-transcriptional activity towards STAT5B. The precise contribution of the aberrant Y682 phosphorylation in STAT5A, however, remains to be determined. In addition to aberrant phopshorylation, the abundance of STAT5 isoforms and that of other signalling molecules and their tyrosine phosphorylation status may impact on the formation of cytoplasmic pSTAT5-complexes in a cell line and cell-lineage specific manner. Such cytoplasmic pSTAT5 complexes may induce specific effects such as an increase in ROS production. Walsch et al. recently described a STAT5A and ABL-tyrosine kinase dependent increase in ROS production and a highly significant and clinically relevant correlation between STAT5A expression and mutation status of BCR-ABL [37]. Although the functional impact of cytoplasmic pSTAT5 complexes remains to be precisely characterized the data suggest different functions for STAT5A and STAT5B in the context of BCR-ABL which are, however, both suitable for specific therapeutic intervention.

## Supporting Information

Figure S1 **Time and dose dependent expression of BCR-ABL in TonB cells.**
(DOC)

Figure S2 **TonB cell apoptosis in the presence of STAT5A and STAT5B specific shRNAs.**
(DOC)

Figure S3 **Tyrosine phosphorylation of STAT5B in TonB cells upon STAT5B over-expression.**
(DOC)

Figure S4 **Homodi-(oligo)merization of STAT5A and STAT5B in TonB cells.**
(DOC)

Figure S5 **Overview of putative mass to charge ratios of the identified STAT5A phosphopeptide.**
(DOC)

Figure S6 **Mutant analysis of YYTPVLAK and identification of the phosphorylation event in PBMCs from a patient with first diagnosed CML.**
(DOC)

Figure S7 **Putative mass spectrometric values for tandem MS-generated product ions from the tryptic peptides FYTPVLAK, YFTPVLAK emanating from mutated STAT5A.**
(DOC)

Figure S8 **Coimmunoprecipitates with wildtype and mutant STAT5A.**
(DOC)

Figure S9 **Effects of isoform-specific STAT5 shRNAs on primary CD34^+^ cells.**
(DOC)

Information S1 **Supplementary Materials and Methods.**
(DOC)
