# Supplementary material for: BCR-ABL Affects STAT5A and STAT5B Differentially
Source: PLoS One. 2014 May 16;9(5):e97243. doi: 10.1371/journal.pone.0097243 (PMC4023949; doi:10.1371/journal.pone.0097243)
Supplement: Figure S1 — Time and dose dependent expression of BCR-ABL in TonB cells. (DOC) [file pone.0097243.s001.doc]

**Supplementary Figure S1**


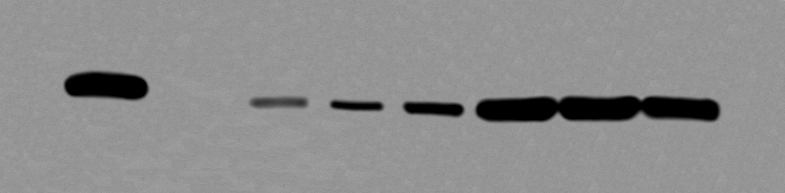

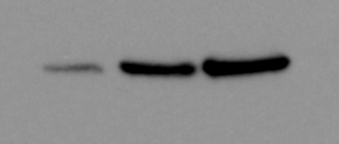

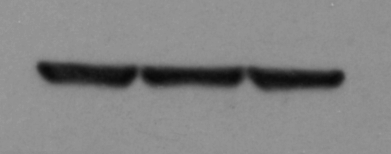

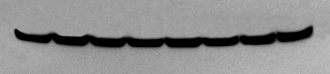


K562

TonB + BCR-ABL

[**hours of stimulation with**

**2.0 µg/mL doxycycline**]

0 1 3 6 24 48 72 0.2 1.0 2.0

**IB:** c-ABL

**IB:** Actin

TonB + BCR-ABL

[**µg/mL doxycycline**]

250 kDa

37 kDa

50 kDa

100 0 11 18 29 87 83 66 [%]

**Quantification:**

**Supplementary Figure S1: Time and dose dependent expression of BCR-ABL in TonB cells.**

TonB cell cultures were depleted of IL-3 and doxycycline was added to induce BCR-ABL expression as indicated. Cells were lysed and analyzed by western blotting for BCR-ABL-expression using c-ABL and Actin (loading control) specific antibodies. BCR-ABL-expression of TonB cells was quantified in comparison to that in K562 cells by densitometry.
