# Supplementary material for: BCR-ABL Affects STAT5A and STAT5B Differentially
Source: PLoS One. 2014 May 16;9(5):e97243. doi: 10.1371/journal.pone.0097243 (PMC4023949; doi:10.1371/journal.pone.0097243)
Supplement: Figure S2 — TonB cell apoptosis in the presence of STAT5A and STAT5B specific shRNAs. (DOC) [file pone.0097243.s002.doc]

**Supplementary Figure S2**

**IL-3 [72 hours] BCR-ABL [72 hours]**


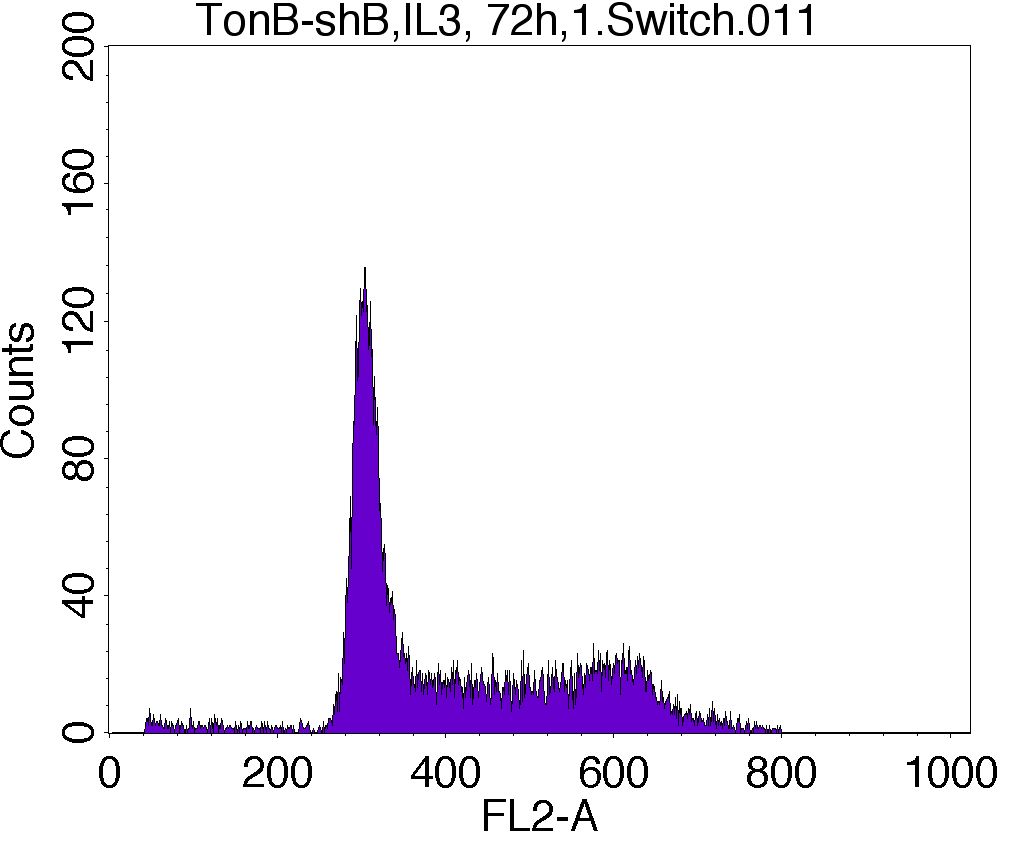


Sub-G1


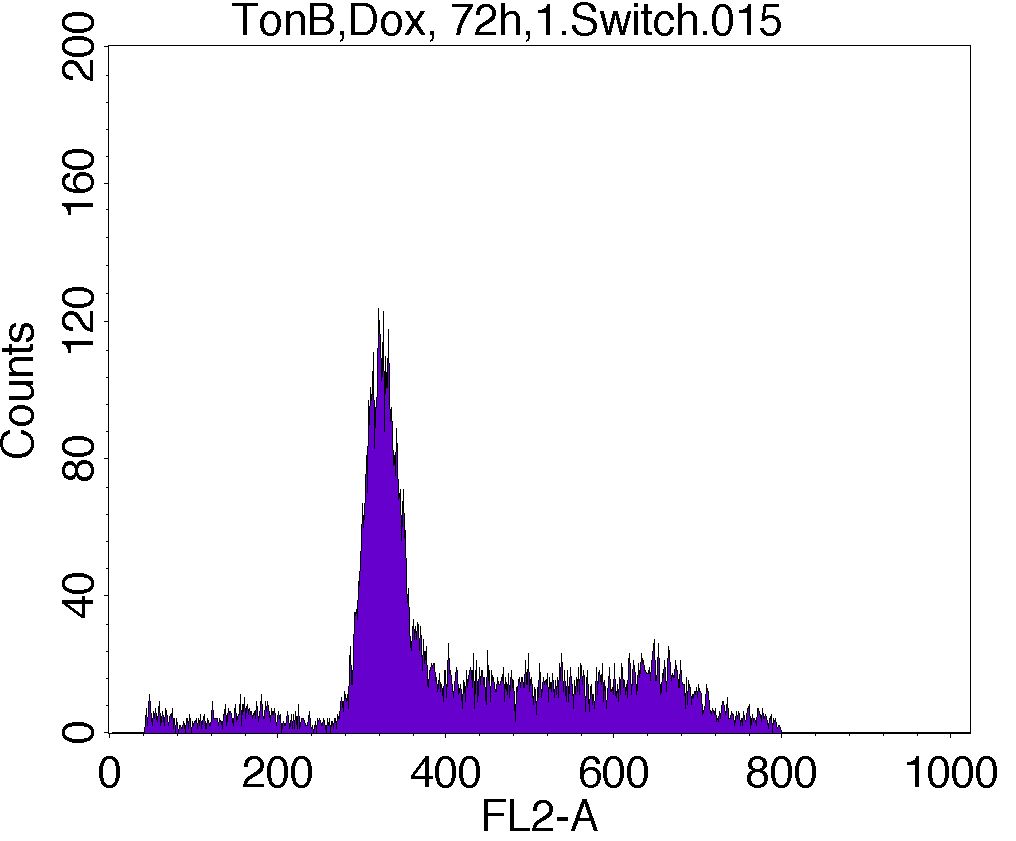


Sub-G1

**TonB**


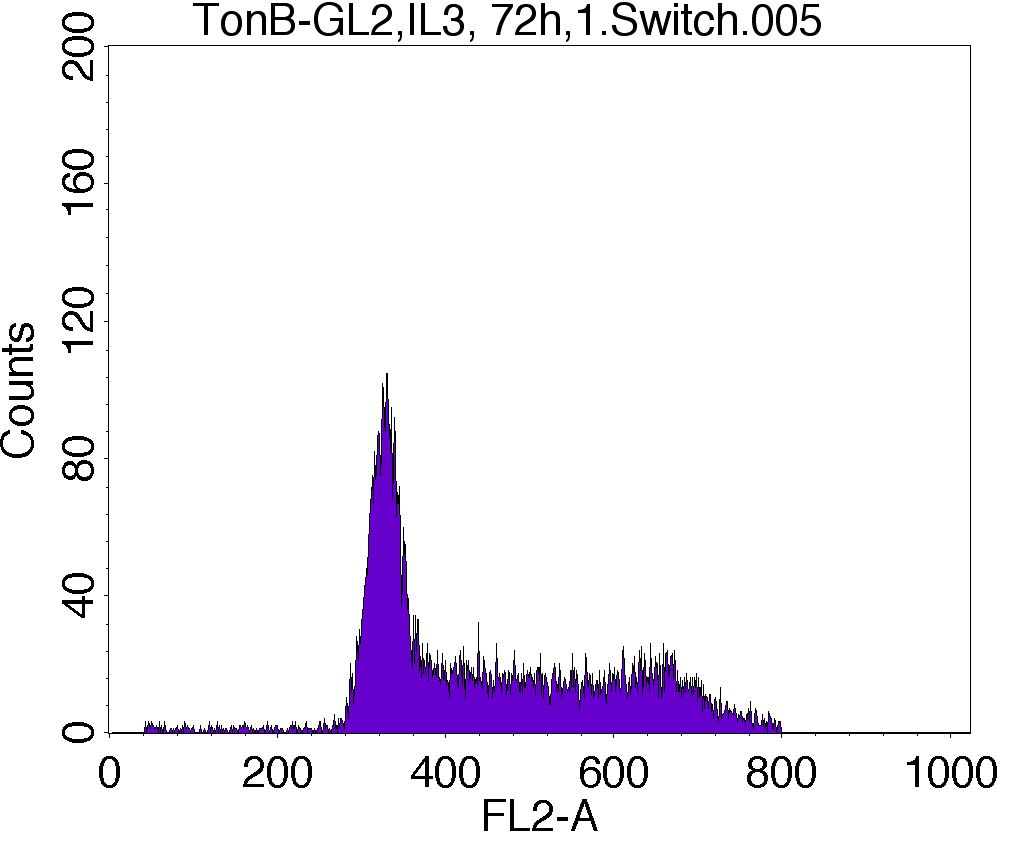


Sub-G1


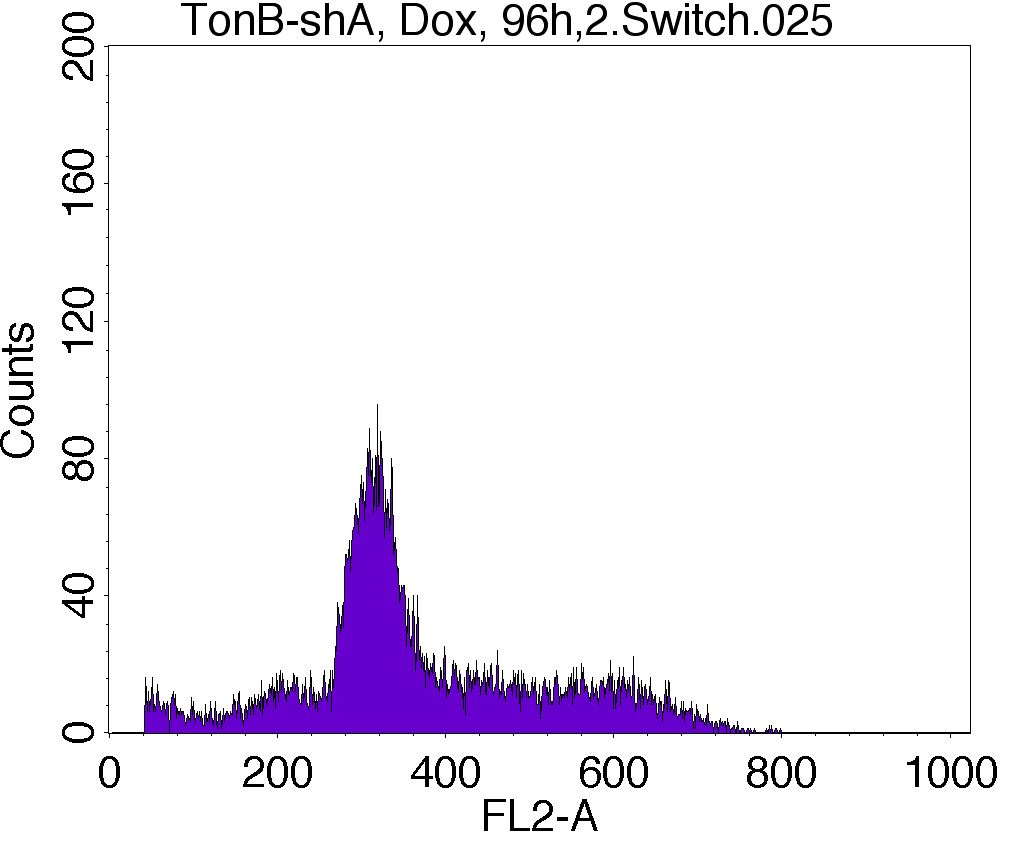


Sub-G1

**TonB + shGL2-ctrl**


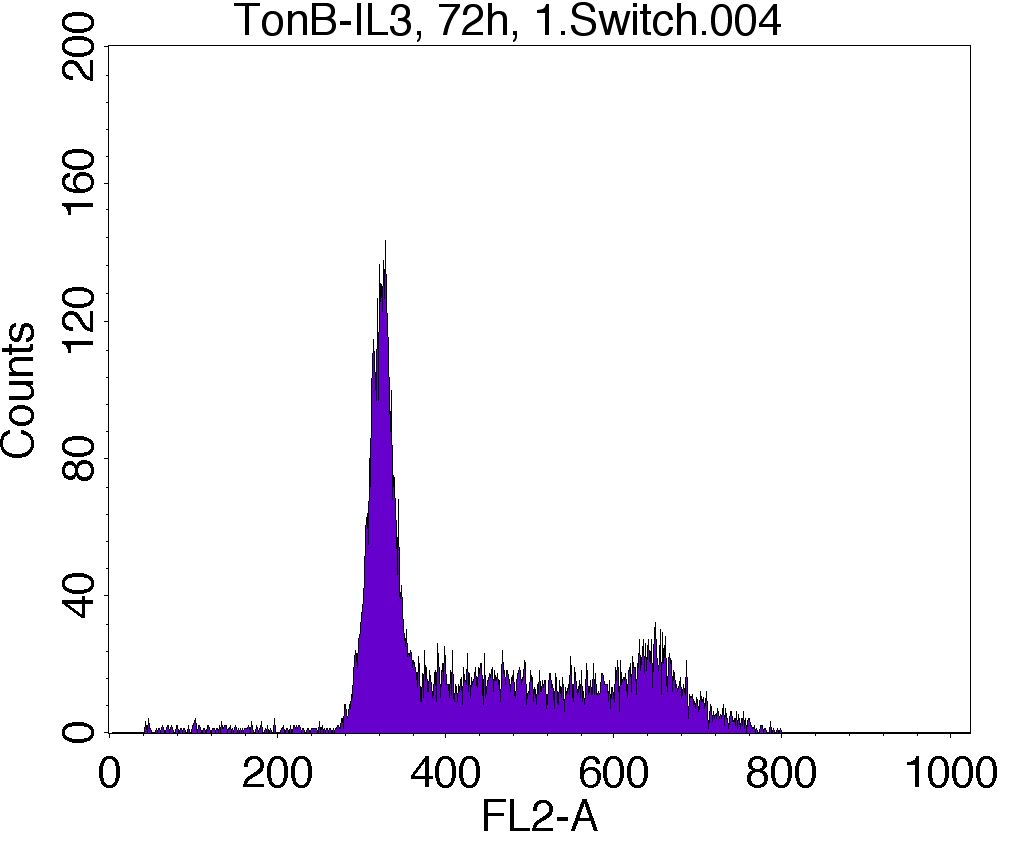


Sub-G1


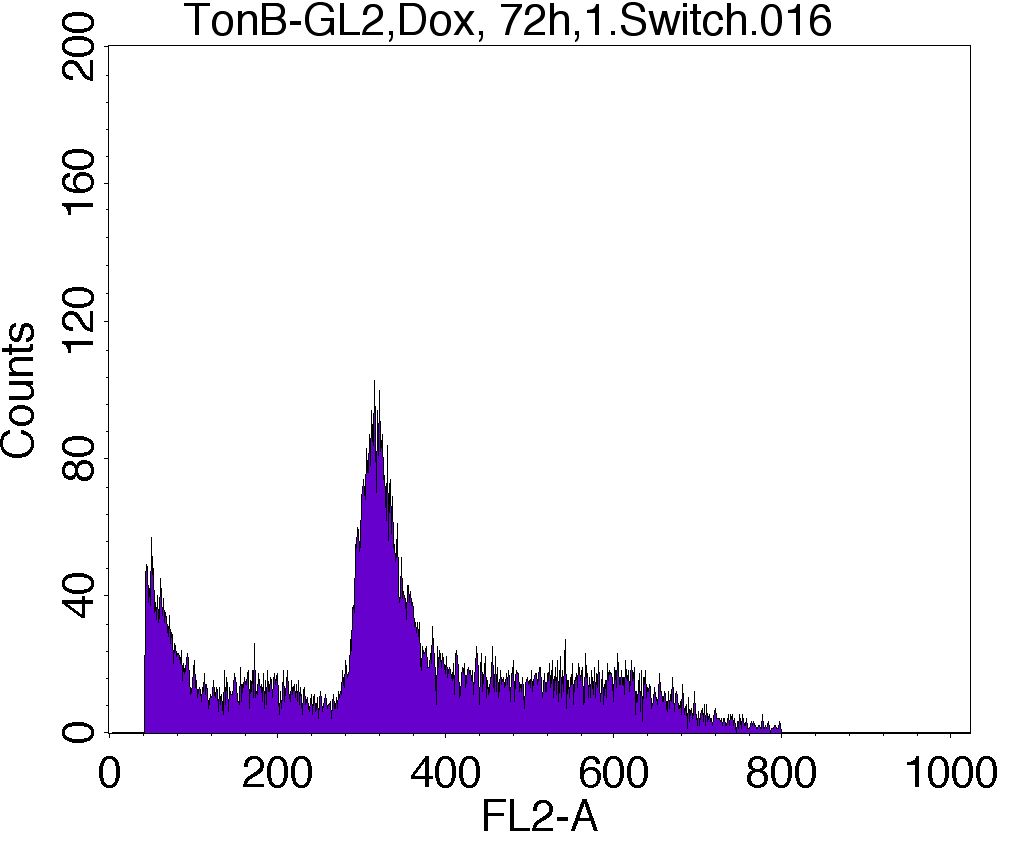


Sub-G1

**TonB + sh-muS5A**


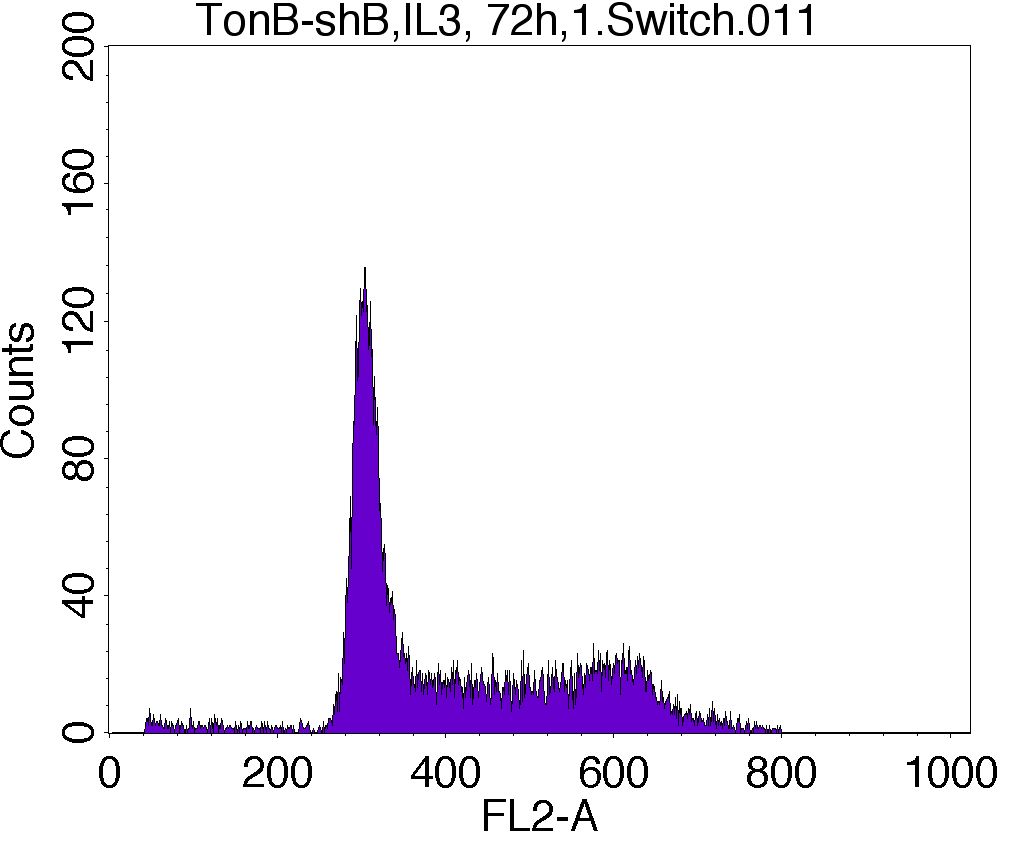


Sub-G1


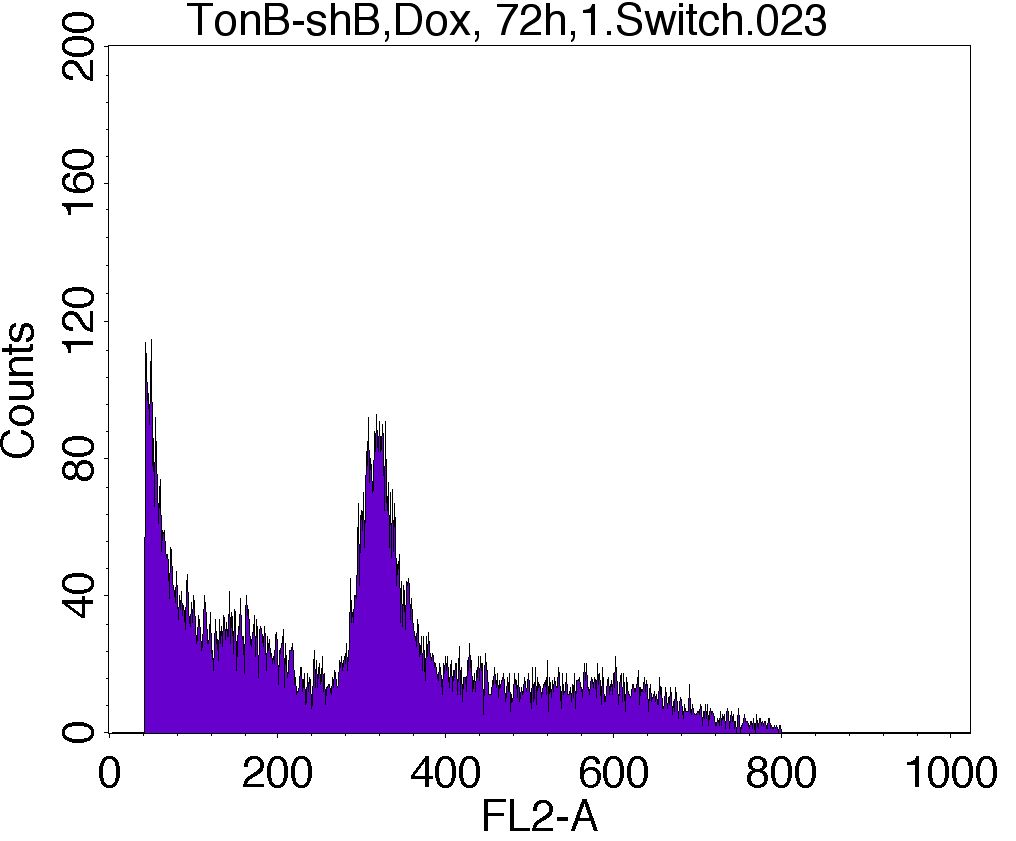


Sub-G1

**TonB + sh-muS5B**

**Supplementary Figure S2: TonB cell apoptosis in the presence of STAT5A and STAT5B specific shRNAs.**

TonB cells were lentivirally transduced to express GL-2 control (shGL2-ctrl), murine STAT5A (sh-muS5A) or murine STAT5B (sh-muS5B) shRNAs. Three days after transduction cells were cultured in IL-3 or 1.5 µg/mL doxycycline for induction of BCR-ABL-expression. After 72 hours cells were fixed with Methanol (80% v/v) and stored three days at 2-8°C for degradation of lentivirally co-expressed RFP. After Propidium iodide and RNaseA treatment cells were analysed by FACS. Representative cell cycle distributions are shown. Amounts of cells in Sub-G1 phase were quantified.
