# Supplementary material for: BCR-ABL Affects STAT5A and STAT5B Differentially
Source: PLoS One. 2014 May 16;9(5):e97243. doi: 10.1371/journal.pone.0097243 (PMC4023949; doi:10.1371/journal.pone.0097243)
Supplement: Figure S3 — Tyrosine phosphorylation of STAT5B in TonB cells upon STAT5B over-expression. (DOC) [file pone.0097243.s003.doc]

**Supplementary Figure S3**

**Supplementary Figure S3: Tyrosine phosphorylation of STAT5B in TonB cells upon STAT5B over-expression.**

TonB cells were lentivirally transduced to over-express wildtype STAT5B and cultured in the absence of IL-3 and doxycycline. Whole cell lysates were prepared at day 2, 9 and 13 of starvation and tyrosine phosphorylation of STAT5B- and BCL-XL-expression were analyzed by western blotting.
