# Supplementary material for: BCR-ABL Affects STAT5A and STAT5B Differentially
Source: PLoS One. 2014 May 16;9(5):e97243. doi: 10.1371/journal.pone.0097243 (PMC4023949; doi:10.1371/journal.pone.0097243)
Supplement: Figure S4 — Homodi-(oligo)merization of STAT5A and STAT5B in TonB cells. (DOC) [file pone.0097243.s004.doc]

**Supplementary Figure S4**

**A**

**‡ ± ‡ ‡ + ±**

Myc-

Tag

**IB:** Myc-Tag

**IB:** HA-Tag

**IB:** pY694/699


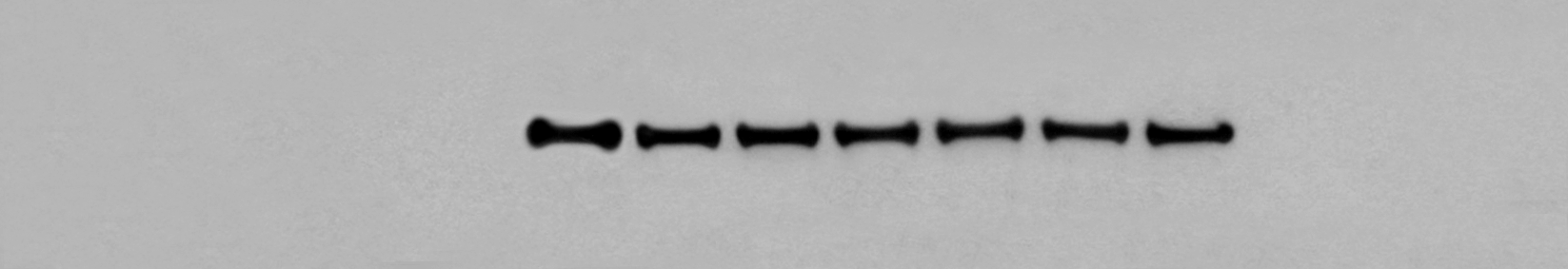

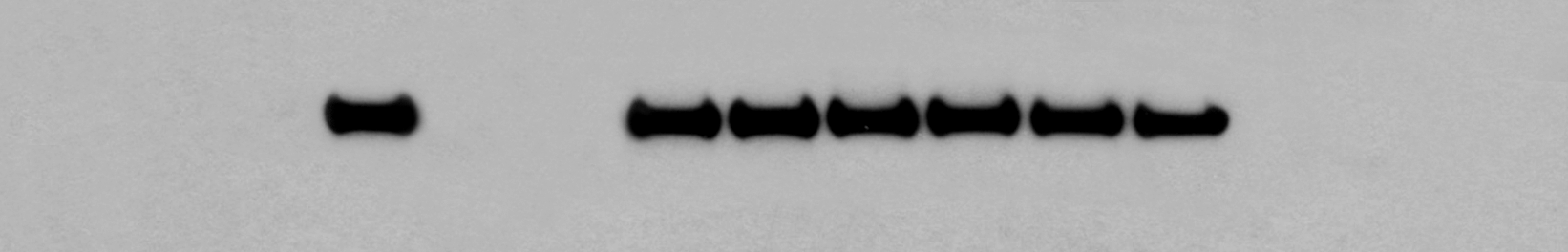


Myc-

Tag

HA-

Tag

TonB

STAT5A-**MYC**

TonB

STAT5A-**HA**

IL-3

Starvation

Restimulation

IL-3+BCR-ABL

BCR-ABL

BCR-ABL+IM

A-**Myc +** A-**HA**


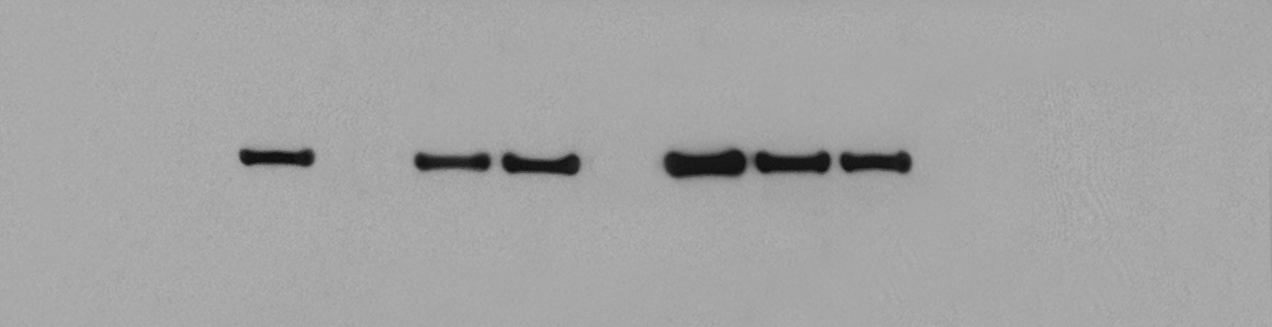

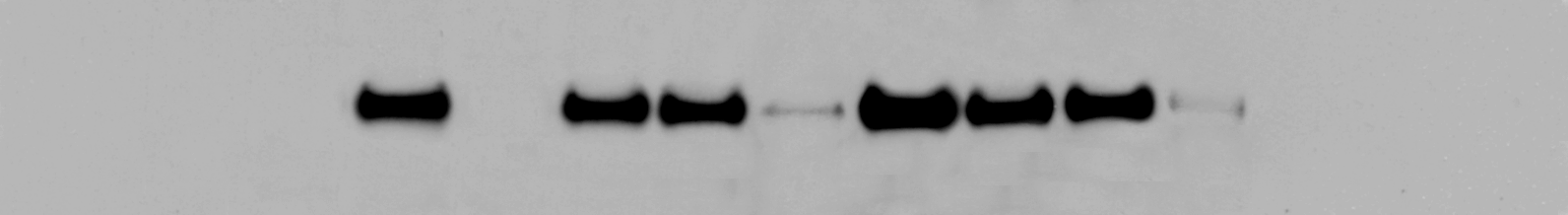

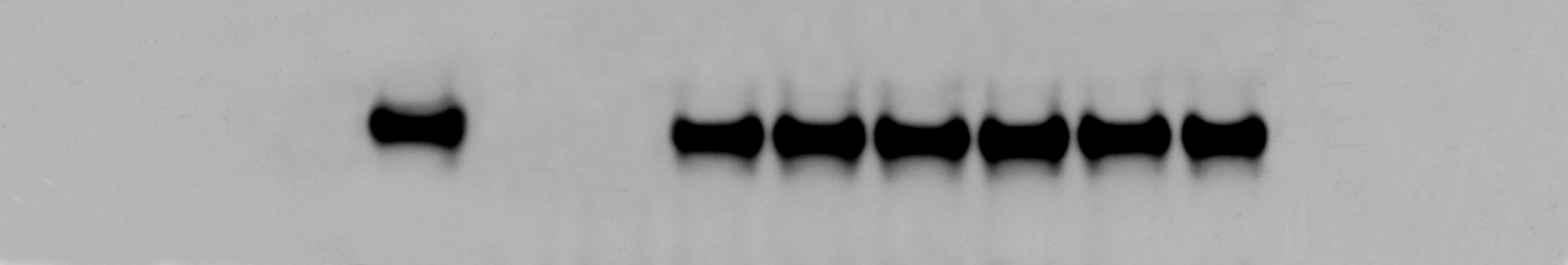

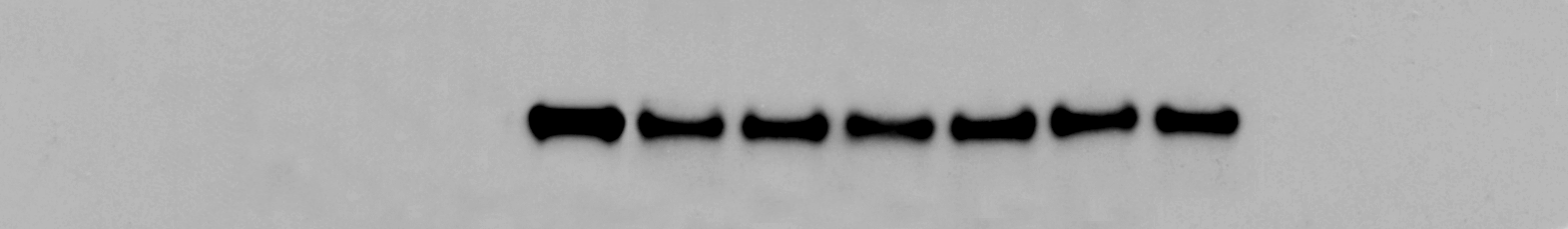


Myc-

Tag

B-**Myc +** B-**HA**

HA-

Tag

Myc-

Tag

TonB

STAT5B-**MYC**

TonB

STAT5B-**HA**

IL-3

Starvation

Restimulation

IL-3+BCR-ABL

BCR-ABL

BCR-ABL+IM

- 100 K

- 75 K

- 100 K

- 75 K

- 100 K

- 75 K

**Viability: ‡ o ‡ ‡ + o**

**IP:**

# B

**IP:**

IL-3

Starvation

**IB:** HA-Tag

**IB:** Myc-Tag

**IB:** pY694/699

B-**Myc +** B-**HA**

RIPA

CF

NF

5x NF

RIPA

CF

NF

5x NF

IL-3

Starvation

IL-3

Starvation

IL-3

Starvation

IL-3

Starvation

IL-3

Starvation

IL-3

Starvation

IL-3

Starvation


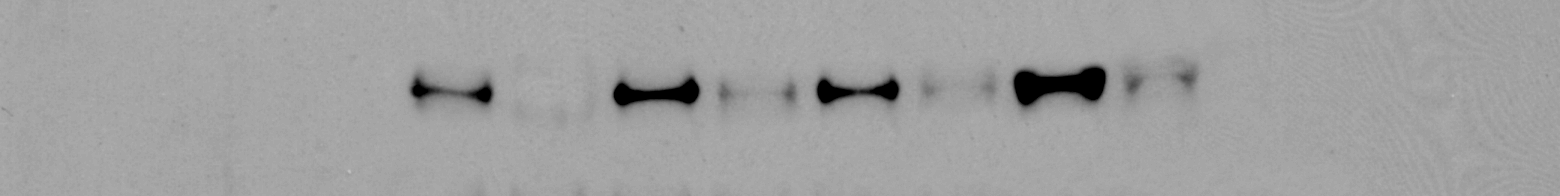

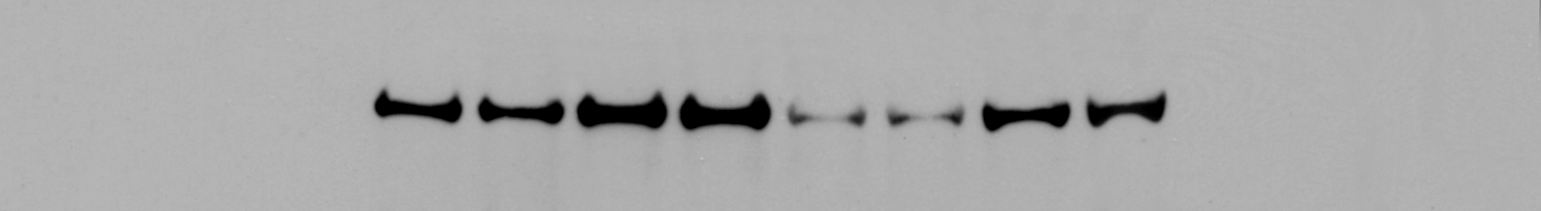

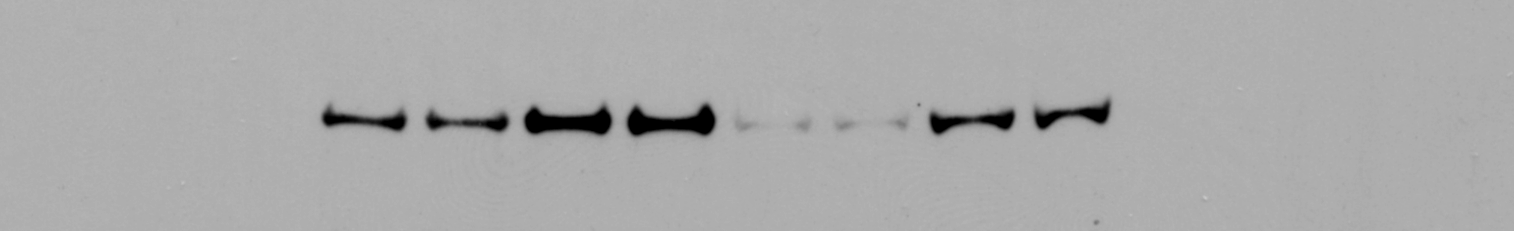

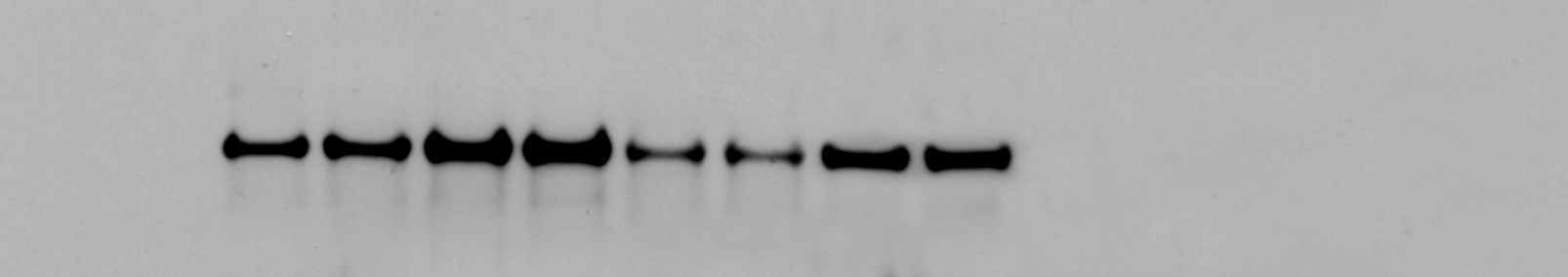

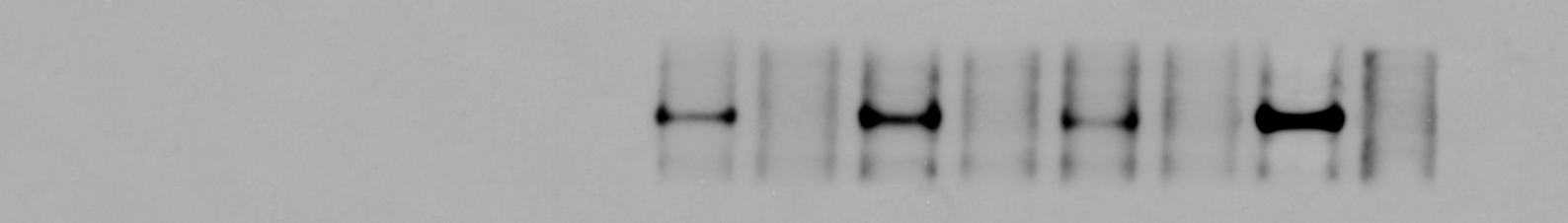

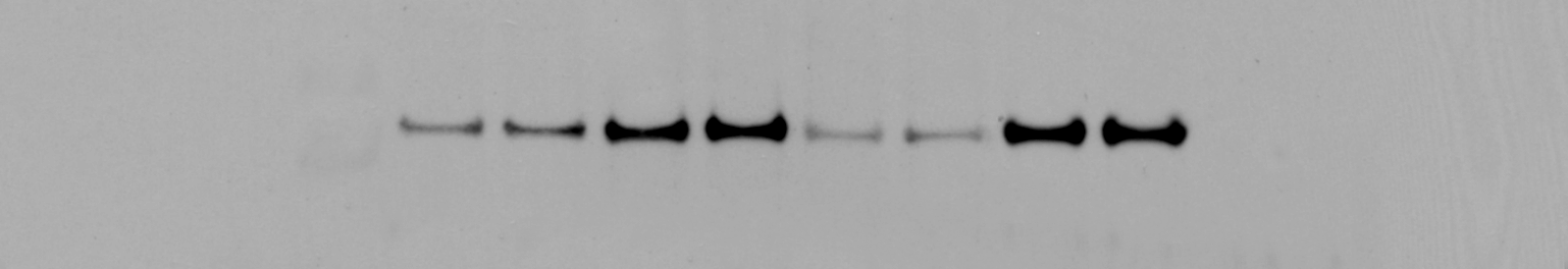


A-**Myc +** A-**HA**

HA-Tag

- 100 K

- 75 K

- 100 K

- 75 K

- 100 K

- 75 K

**Supplementary Figure S4: Homodi-(oligo)merization of STAT5A and STAT5B in TonB cells**

(A) TonB cells lentivirally transduced with one or two differentially tagged variants (HA and MYC) of STAT5A (left) and STAT5B (right), respectively, were cultured in the presence of IL‑3, BCR‑ABL, and IL‑3 plus BCR‑ABL. Aliquots were starved overnight by withdrawal of IL‑3 (Starvation) and re-stimulated with IL‑3 for 30 minutes (Restimulation) or were treated with 1 µM imatinib mesylate (BCR‑ABL+IM) as described for Figure 5. The left four lanes in both panels show transgenic expression of the respective epitope-tagged STAT5 isoform in IL‑3 cultures. One variant of tagged STAT5A (left) or tagged STAT5B (right) was immunoprecipitated by the indicated antibodies. Co-precipitation of the alternatively tagged STAT5 isoform and phosphorylation of the critical tyrosine residue were analyzed by western blotting. Viability was measured at day 3 by Trypan-blue dye exclusion assay and classified as follows: ‡: ≥ 75%; +: ≥ 50%; ±: ≥ 25%; o: ≤ 10% viable cells.

(B) TonB cells co-expressing STAT5A-HA + STAT5A-MYC (left) or STAT5B-HA + STAT5B-MYC (right) were either cultured in the presence of IL‑3 or starved by withdrawal of IL‑3 overnight. Whole cell lysates (RIPA) as well as cytoplasmic (CF) and nuclear (NF) extracts were prepared and HA-tagged STAT5A (left) and STAT5B (right) were precipitated, analyzed for co-precipitation of the MYC-tagged counter­parts and phosphorylation of the critical tyrosine residue by western blotting. Data represent mean of three independent experiments.
