# Supplementary material for: BCR-ABL Affects STAT5A and STAT5B Differentially
Source: PLoS One. 2014 May 16;9(5):e97243. doi: 10.1371/journal.pone.0097243 (PMC4023949; doi:10.1371/journal.pone.0097243)
Supplement: Figure S5 — Overview of putative mass to charge ratios of the identified STAT5A phosphopeptide. (DOC) [file pone.0097243.s005.doc]

**Supplementary Figure S5**

B ion

Y ion

---

Y

---

327.1339

Y

791.4662

428.1816

T

628.4028

525.2344

P

527.3552

624.3028

V

430.3024

737.3869

L

331.2340

808.4240

A

218.1499

---

K

147.1128

---

B ion

407.1003

508.1479

605.2007

704.2691

817.3532

888.3903

---

K

A

L

V

P

T

Y

pY

Amino

Acid

Y ion

---

**791.4662**

628.4028

**527.3552**

430.3024

331.2340

218.1499

147.1128

B ion

Y ion

---

Y

---

407.1003

pY

**871.4325**

508.1479

T

628.4028

605.2007

P

**527.3552**

704.2691

V

430.3024

817.3532

L

331.2340

888.3903

A

218.1499

---

K

147.1128

Amino

Acid

Amino

Acid

**Supplementary Figure S5: Overview of putative mass to charge ratios of the identified STAT5A phosphopeptide**

The figure shows the putative mass to charge ratios (m/z) of the ions for the peptide YYTPVLAK derived from STAT5 and also for its phosphorylated form (phosphate on the first or second tyrosine residue). The b ion and y ion header refers to the type of fragment ion detected by the mass spectrometer. Ions with the parent mass/charge ratios of 477.77Th (unphosphorylated) and 517.75Th (phosphorylated) were selected for tandem mass spectrometric analysis. Specific product ions from the fragmented parent ion were selected to permit verification of the ion identity and to confirm the site of phosphorylation. The bold numbers refer to these ions: 527.3552Th identifies the peptide; 871.4325Th, 791.4662Th and 216.04Th identify the site of phosphory­lation. The 216.04Th ion is diagnostic for tyrosine phosphorylation.
