# Supplementary material for: BCR-ABL Affects STAT5A and STAT5B Differentially
Source: PLoS One. 2014 May 16;9(5):e97243. doi: 10.1371/journal.pone.0097243 (PMC4023949; doi:10.1371/journal.pone.0097243)
Supplement: Figure S6 — Mutant analysis of YYTPVLAK and identification of the phosphorylation event in PBMCs from a patient with first diagnosed CML. (DOC) [file pone.0097243.s006.doc]

**Supplementary Figure S6**

**A**

**TonB, BCR-ABL**

**STAT5A-Y682F *– FY****TPVLAK*

***FpY****TPVLAK*

**B**

**TonB, BCR-ABL**

***pYF****TPVLAK*

**STAT5A-Y683F *– YF****TPVLAK*

**C**

**TonB, BCR-ABL**

**STAT5A-Y682/683F *– FF****TPVLAK*

**No phosphorylated parent ion measurable!**

ion chromatogram for m/z 791Th (indicative of **pY**YTPVLAK)

**D**

**CML-patient**

Time (minutes)


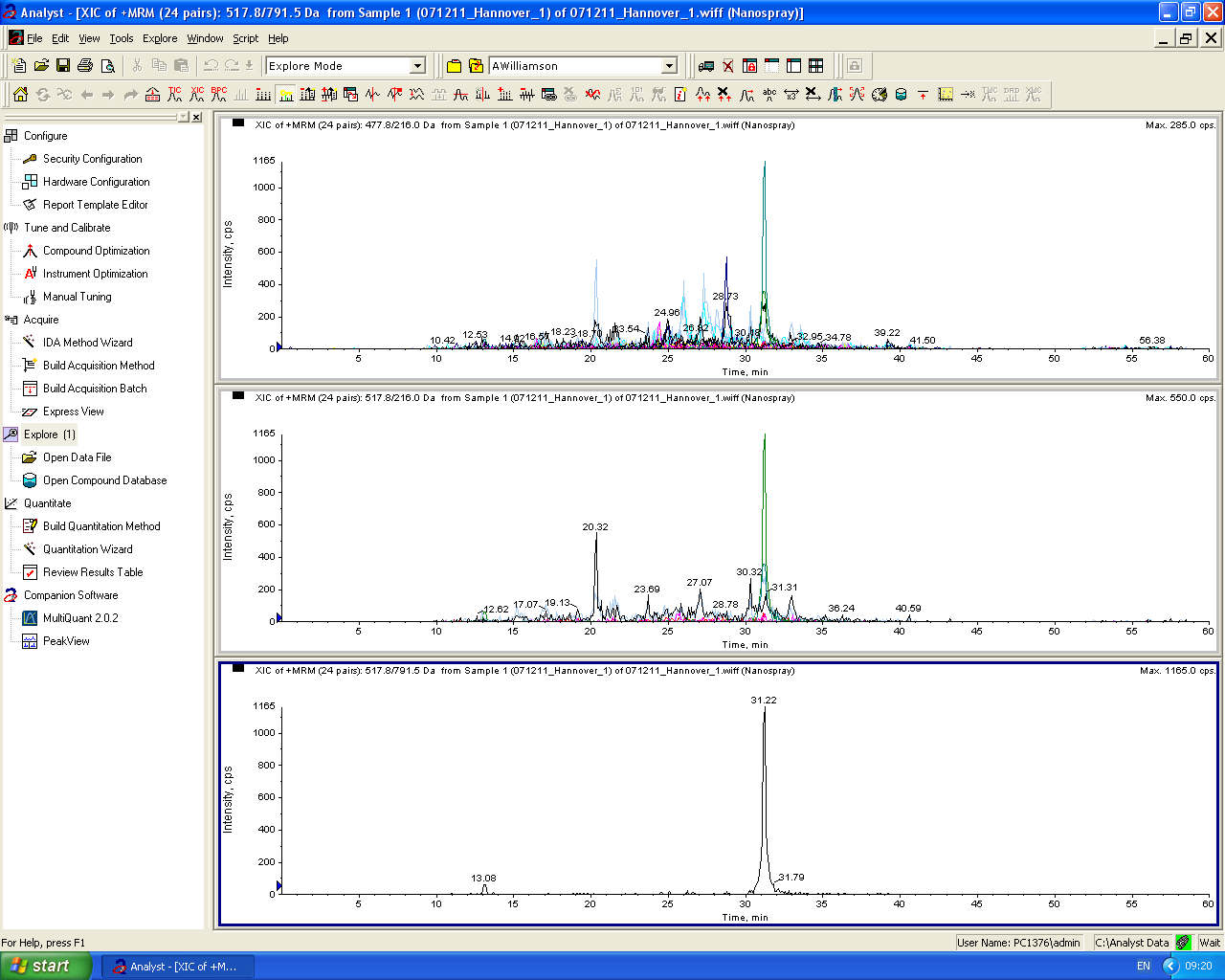


***pY****YTPVLAK* – Parent ion: 517.75Th; Product ion: 791.47Th

**Supplementary Figure S6: Mutant analysis of YYTPVLAK and identification of the phosphorylation event in PBMCs from a patient with first diagnosed CML**

The figure shows the putative mass to charge ratios (m/z) of the ions for the mutated peptide; Y682F, Y683F and Y682/683F and also for its phosphorylated form. Specific product ions from the fragmented parent ion were selected to permit verification of the ion identity and to confirm the site of phosphorylation. For further information of these ions see Supplementary Figure S7. (A-C) Results from the SRM analysis of the Y to F mutants confirming Y682 as the site of phosphorylation. (D) Identification of the phosphorylation event in CML-patient material. The 4000 QTrap (AB Sciex) was utilized. In this analysis the shown panel identifies a product ion of 791.47Th from the parent ion of 517.75Th. For further information of the mentioned ions see Supplementary Figure S5.
