# Supplementary material for: BCR-ABL Affects STAT5A and STAT5B Differentially
Source: PLoS One. 2014 May 16;9(5):e97243. doi: 10.1371/journal.pone.0097243 (PMC4023949; doi:10.1371/journal.pone.0097243)
Supplement: Figure S7 — Putative mass spectrometric values for tandem MS-generated product ions from the tryptic peptides FYTPVLAK, YFTPVLAK emanating from mutated STAT5A. (DOC) [file pone.0097243.s007.doc]

**Supplementary Figure S7**

Amino Acid

Amino Acid

B ion

Y ion

B ion

Y ion

---

F

---

---

F

---

311.1390

Y

**791.4662**

391.1053

**pY**

**871.4325**

412.1867

T

628.4028

**492.1530**

T

628.4028

509.2395

P

**527.3552**

589.2058

P

**527.3552**

608.3079

V

430.3024

688.2742

V

430.3024

721.3919

L

331.2340

801.3583

L

331.2340

792.4291

A

218.1499

872.3954

A

218.1499

---

K

147.1128

---

K

147.1128

Amino Acid

Amino Acid

B ion

Y ion

B ion

Y ion

---

Y

---

---

**pY**

---

311.1390

F

775.4713

391.1053

F

775.4713

412.1867

T

628.4028

509.2395

P

**527.3552**

608.3079

V

430.3024

721.3919

L

331.2340

792.4291

A

218.1499

---

K

147.1128

**492.1530**

T

628.4028

589.2058

P

**527.3552**

688.2742

V

430.3024

801.3583

L

331.2340

872.3954

A

218.1499

---

K

147.1128

**Supplementary Figure S7: Putative mass spectrometric values for tandem MS-generated product ions from the tryptic peptides FYTPVLAK, YFTPVLAK emanating from mutated STAT5A.**

The table lists the product ions from the mutated STAT5A peptides, FYTPVLAK, YFTPVLAK and their putative phosphorylated forms. The b ion and y ions refer to the type of fragment ion detected by the mass spectrometer in a tandem mass spectrometry experiment. The bold numbers refer to the tandem MS ions used to infer identity and phosphorylation status. 527.3552Th identifies the peptide, 871.4325Th, 791.4662Th and 492.1530Th identify the site of phosphorylation.
