# Supplementary material for: BCR-ABL Affects STAT5A and STAT5B Differentially
Source: PLoS One. 2014 May 16;9(5):e97243. doi: 10.1371/journal.pone.0097243 (PMC4023949; doi:10.1371/journal.pone.0097243)
Supplement: Figure S8 — Coimmunoprecipitates with wildtype and mutant STAT5A. (DOC) [file pone.0097243.s008.doc]

**Supplementary Figure S8**

**TonB+sh-muS5A + pS-S5A-IEW**

**IP: STAT5A**

**IB:** p-Tyrosine


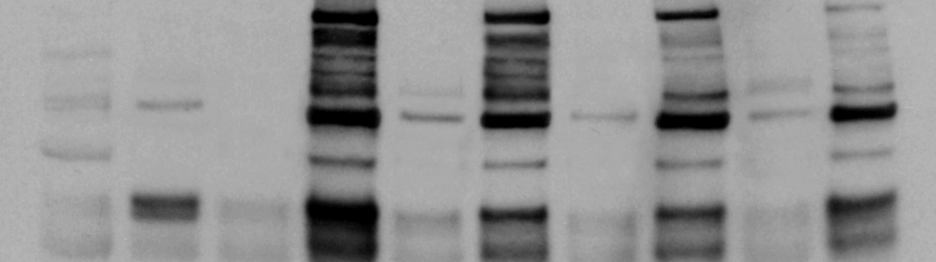


*p-Tyr-STAT5A*


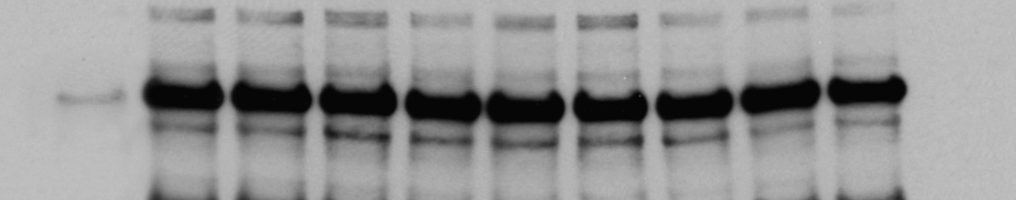


**IB:** STAT5A

**Supplementary Figure S8: Coimmunoprecipitates with wildtype and mutant STAT5A**

A TonB cell clone with no detectable endogenous STAT5A expression (due to anti- STAT5A RNAi) (ctrl, Lane 1) was transduced with lentiviruses encoding STAT5A-Wildtype (WT) or STAT5A tyrosine mutants (Y682F; Y683F; Y682/83F). Cells were cultured in the presence of IL-3 or doxycycline (1.5 µg/mL) for induction of BCR-ABL-expression. Whole cell lysates were prepared and STAT5A was immunoprecipitated. Tyrosine phosphorylation of STAT5A and proteins co-precipitated with STAT5A was analyzed by western blotting.
