# Supplementary material for: BCR-ABL Affects STAT5A and STAT5B Differentially
Source: PLoS One. 2014 May 16;9(5):e97243. doi: 10.1371/journal.pone.0097243 (PMC4023949; doi:10.1371/journal.pone.0097243)
Supplement: Figure S9 — Effects of isoform-specific STAT5 shRNAs on primary CD34+ cells. (DOC) [file pone.0097243.s009.doc]

**Supplementary Figure S9**

| **Table 2. Effects of lentivirally encoded shRNAs on colony formation of CML and normal CD34^+^ cells** | | | | | |
| --- | --- | --- | --- | --- | --- |
| Sample,  dcH1-shRNA-SR | Total colony number | Number of RFP^+^ colonies | Ratio of RFP^+^ colonies to total colony number [%] | Effect of  STAT5 inhibition in comparison to GL2 ctrl. cells [%] | RFP expression in suspension cultures  [%] |
| **CML CD34^+^ cells** |  |  |  |  |  |
| **A** GL2 | 86 | 26 | 30 | **100** | 27 |
| huSTAT5A | 78 | 28 | 36 | 120 | 36 |
| huSTAT5B | 122 | 34 | 28 | 93 | 30 |
| **B** GL2 | 37 | 30 | 81 | **100** | 76 |
| huSTAT5A | 21 | 12 | 57 | 70 | 72 |
| huSTAT5B | 34 | 24 | 70 | 86 | 77 |
| **C** GL2 | 131 | 72 | 55 | **100** | 53 |
| huSTAT5A | 161 | 83 | 52 | 95 | 66 |
| huSTAT5B | 65 | 34 | 52 | 95 | 57 |
| **D** GL2 | 56 | 40 | 71 | **100** | 70 |
| huSTAT5A | 76 | 42 | 55 | 77 | 67 |
| huSTAT5B | 62 | 38 | 61 | 86 | 72 |
| **Normal CD34^+^ cells** |  |  |  |  |  |
| **A** GL2 | 25 | 6 | 24 | **100** | 8 |
| huSTAT5A | 35 | 7 | 20 | 83 | 9 |
| huSTAT5B | 30 | 5 | 17 | 71 | 10 |
| **B** GL2 | 28 | 3 | 11 | **100** | 11 |
| huSTAT5A | 20 | 2 | 10 | 91 | 10 |
| huSTAT5B | 27 | 5 | 19 | 173 | 9 |

**Supplementary Figure S9: Effects of isoform-specific STAT5 shRNAs on primary CD34^+^ cells**

CD34^+^ cells from healthy donors and patients at first diagnosis of chronic phase CML were isolated as described [48] and transduced with lentiviral supernatants encoding control (GL2) or isoform-specific STAT5 shRNAs. Between 250 and 1000 cells were plated into methyl cellulose assays in the presence of 20 ng/mL GM-CSF and 10 mg/mL IL‑3. Transduction efficacy was determined in suspension cultures 5 days after transduction by FACS analysis. Colonies were counted at day 11, and number of RFP^+^ colonies was divided by total colony count. Relative inhibition of colony formation by each condition and transduction efficacy of cells in suspension cultures are also indicated.
