# Supplementary material for: BCR-ABL Affects STAT5A and STAT5B Differentially
Source: PLoS One. 2014 May 16;9(5):e97243. doi: 10.1371/journal.pone.0097243 (PMC4023949; doi:10.1371/journal.pone.0097243)
Supplement: Information S1 — Supplementary Materials and Methods. (DOC) [file pone.0097243.s010.doc]

Supplementary information:

**Materials and Methods:**

*shRNA synthesis*

The cDNA sequences (CDS) of murine STAT5 isoforms (STAT5A: GenBank accession no. NM_011488.3; STAT5B: GenBank accession no. NM_011489.3) or of human STAT5 isoforms (STAT5A: GenBank accession no. NM_003152.3; STAT5B: GenBank accession no. NM_012448.3) were aligned. Heterogeneous sequences between STAT5A and STAT5B were used for the design of isoform-specific STAT5 shRNAs. For murine and human STAT5 five and three shRNAs were selected for each STAT5 isoform, respectively, and subjected to a BLAST-homology search. DNA oligonucleotides were chemically synthesized including overhang sequences from a 5’BglII and a 3’SalI restriction site for cloning purposes (BioSpring, Frankfurt, Germany). Corresponding oligonucleotides were annealed and inserted 3’ of the H1-RNA promoter into a 5’BglII/3’SalI-digested pBlueScript-derived pH1-plasmid. STAT5 shRNAs were functional evaluated as described earlier (24). For each STAT5 isoform one shRNA was found which reduces protein by >90%. The corresponding oligonucleotide sequences are as follows: FP_muS5A: 5’-gatccccgacgcgagatttctccatttcaagagaaatggagaaatctcgcgtcttttttggaag-3’; RP_muS5A: 5’-tcgacttccaaaaaagacgcgagatttctccatttctcttgaaatggagaaatctcgcgtcggg-3’; FP_muS5B: 5’-gatcccccactagagacttctctatcttcaagagagatagagaagtctctagtgttttttggaag-3’; RP_muS5B: 5’-tcgacttccaaaaaacactagagacttctctatctctcttgaagatagagaagtctct­agtgggg-3’; FP_huS5A: 5’-gatccccgtactacactcctgtgctgtggatccacagcacag­gagtgtagtactttt­ttggaag-3’; RP_huS5A: 5’-tcgacttccaaaaaagtactacactcctgtgctgtgg­atccacagc­acagga­­gtgtagtacggg-3’; FR_huS5B: 5’-gatccccgcatgggactcagtagatcttcaa­gagagatctactgagtcccatgcttttttggaag-3’; RP_huS5B: 5’-tcgacttccaaaaaagcatggg­actcagtagatctctcttgaagatctactgagtcccatgcggg-3’. The non-complementary 9-nt-loop sequences are underlined. The six T-nucleotides in sense orientation serve as polymerase III transcription termination signal.

*Immunoblotting and Immunoprecipitation*

Subcellular extracts were prepared by sequential incubation in low salt buffer (20 mM HEPES, pH 7.5; 10 mM KCl; 1 mM MgCl_2_; 0.1% Triton X-100; 20% glycerol; 1 mM DTT) and high salt buffer (20 mM HEPES, pH 7.5; 400 mM NaCl; 1 mM EDTA; 20% glycerol; 0.1% Triton X-100; 1 mM DTT) each supplemented with inhibitors as described in the materials and methods section.

*Construction of lentiviral vectors*

Epitope-tagged STAT5 isoforms were generated by in-frame insertion of chemically synthesized DNA oligonucleotides encoding N-EQKLISEEDL-C (MYC-Tag) or N-YPYDVPDYA-C (HA-Tag), an artificial STOP codon and proper overhangs to allow insertion into the extreme C-terminal regions of STAT5A and STAT5B, respectively (BioSpring, Frankfurt, Germany).

## Isolation of STAT5 from BCR-ABL-positive CML cells

Initially, peripheral blood mononuclear cells (PBMCs) from first diagnosed BCR-ABL-positive CML-patients or healthy donors were prepared by density gradient centrifugation using Biocoll Separating Solution (Biochrome, Berlin, Germany). Informed consent was obtained in accordance with the Declaration of Helsinki.

PBMCs were washed twice in phosphate buffered saline and lysed in RIPA buffer. A potential improper ratio of protein inhibitors to RIPA buffer-lysed cells was compensated by a 3-fold excess of inhibitors as described in the materials and methods section. While this approach led to functional protein isolation from PBMCs of healthy donors, no STAT5 proteins were detected by western blotting in lysates from CML-PBMCs. Due to this unexpected high proteolytical activity an alternative protein isolation method was established. Henceforward, PBMCs from two CML-patients were prepared as described above and 1x10^9^ cells were lysed in 50 mL Trizol Reagent (Invitrogen, Karlsruhe, Germany). After clarification 10 mL chloroform was added, the suspension thoroughly mixed and centrifuged (2.000*g*, 4°C, and 5 minutes). The water-soluble upper phase was discarded and 15 mL ethanol was added. After incubation at room temperature (5 minutes) the suspension was centrifuged (2.000*g*, 4°C, and 5 minutes). A 6-fold excess of acetone was added to the phenolic/alcoholic phase. The mixture was inverted several times and incubated at room temperature for 10 minutes. Proteins were precipitated by centrifugation (5000*g*, 4°C, and 10 minutes). Precipitated proteins were washed 3 times in 95% [v/v] ethanol, 2.5% [v/v] glycerol, and 300 mM guanidine hydrochloride. Finally, proteins were air-dried and resolved in 9.5 M urea, 4% [w/v] CHAPS buffer supplemented with protein inhibitors overnight.

In one of two CML-samples phosphorylation at STAT5A-Y682 was measured whereas in the other the amount of precipitated STAT5A protein was too low to detect STAT5 via MS/MS analysis.
